# SOX2 is required independently in both stem and differentiated cells for pituitary tumorigenesis in *p27*-null mice

**DOI:** 10.1073/pnas.2017115118

**Published:** 2021-02-11

**Authors:** Veronica Moncho-Amor, Probir Chakravarty, Christophe Galichet, Ander Matheu, Robin Lovell-Badge, Karine Rizzoti

**Affiliations:** ^a^Laboratory of Stem Cell Biology and Developmental Genetics, The Francis Crick Institute, NW1 1AT London, United Kingdom;; ^b^Bioinformatics Core, The Francis Crick Institute, NW1 1AT London, United Kingdom;; ^c^Cellular Oncology Group, Biodonostia Health Research Institute, E-20014 San Sebastian, Spain;; ^d^CIBER de Fragilidad y Envejecimiento Saludable (CIBERfes), 28029 Madrid, Spain;; ^e^IKERBASQUE, Basque Foundation for Science, 48013 Bilbao, Spain

**Keywords:** pituitary, stem cell, tumorigenesis

## Abstract

P27, a cell cycle inhibitor, is also able to drive repression of *Sox2*. This interaction plays a crucial role during development of *p27*^*−/−*^ pituitary tumors because loss of one copy of *Sox2* impairs tumorigenesis [H. Li *et al.*, *Cell Stem Cell* 11, 845–852 (2012)]. However, SOX2 is expressed in both endocrine and stem cells (SCs), and its contribution to tumorigenesis in either cell type is unknown. We have thus explored the cellular origin and mechanisms underlying endocrine tumorigenesis in *p27*^*−/−*^ pituitaries. We found that pituitary hyperplasia is associated with reduced cellular differentiation, in parallel with increased levels of SOX2 in stem and endocrine cells. Using conditional loss-of-function and lineage tracing approaches, we show that SOX2 is required cell autonomously in *p27*^*−/−*^ endocrine cells for these to give rise to tumors, and in SCs for promotion of tumorigenesis. This is supported by studies deleting the *Sox2* regulatory region 2 (*Srr2*), the target of P27 repressive action. Single cell transcriptomic analysis further reveals that activation of a SOX2-dependent MAPK pathway in SCs is important for tumorigenesis. Altogether, our data highlight different aspects of the role of SOX2 following loss of *p27*, according to cellular context, and uncover an unexpected SOX2-dependent tumor-promoting role for SCs. Our results imply that targeting SCs, in addition to tumor cells, may represent an efficient antitumoral strategy in certain contexts.

## Significance

Tumor development can depend on cell intrinsic dysfunction, but, in some cases, extrinsic factors are important drivers. Here, we established a genetically tractable model, demonstrating that the same gene is relevant both cell autonomously and noncell autonomously for tumorigenesis. Deletion of *p27*, down-regulated in many tumors, predominantly leads to development of murine pituitary tumors. SOX2, transcriptionally derepressed in absence of P27, is important for tumorigenesis in this and other models, but little is known about its interaction. Using loss-of-function and lineage tracing approaches, we establish its regulatory interaction in vivo and show that SOX2 is required independently, both in endocrine and stem cells, to orchestrate tumorigenesis in absence of P27, establishing a powerful model to investigate mechanisms of tumor development.

Tumors frequently originate due to dysfunction of genes acting cell autonomously. However, in rarer cases, cells acquiring mutations may not form the tumor themselves, but instead induce others to do so (1, 2). Regardless of their origin, tumors often display considerable cellular heterogeneity, which can include the presence of cancer stem cells (CSCs), a subpopulation of undifferentiated tumoral cells that may originate from resident tissue stem cells (SCs), and fuel growth and recurrence of the tumor (3). Understanding the precise origins and mechanisms of tumorigenesis is important to decipher therapeutic strategies.

The pituitary is a small endocrine gland, located just under the brain. In the mouse, the anterior lobe (AL) is highly vascularized and contains most endocrine cell populations, while the avascular intermediate lobe (IL) is populated by a single endocrine population, the melanotrophs. These secrete melanocyte stimulating hormone (MSH) which is processed from its precursor pro-opio-melanocortin (POMC), whereas corticotrophs in the AL cleave POMC into adrenocorticotropic hormone (ACTH). Expression of *Pomc* is controlled in both cell types by the T-box factor TBX19 (4). In the embryo, the pioneer transcription factor PAX7 controls acquisition of the melanotroph vs. corticotroph fate (5). Melanotrophs are quiescent, in contrast with AL endocrine cells (6); however, they are particularly sensitive to mutations affecting cell cycle regulators (7). In mice deleted for *p27*, encoding a protein best known for its negative cell cycle regulatory role (8), tumorigenesis in mature animals is the hallmark of its pleiotropic effects (9–11). In the pituitary, this happens specifically in the IL, while the AL is largely unaffected (12). In the developing murine pituitary, P27, along with P57, is necessary for cell cycle exit in differentiating endocrine cells (13). However, its role is not limited to cell cycle regulation, because in *Cyclin D1*^*−/−*^*; p27*^*−/−*^ compound mutants, pituitary hyperplasia is still present (14).

We have previously demonstrated that P27 can recruit cofactors to repress the expression of the transcription factor SOX2 during differentiation of induced pluripotent SCs (iPSCs) (15). SOX2 is an essential factor associated with stemness. It is highly expressed in many cancers and this is associated with poor prognosis (16–18). In vivo, the SOX2-P27 interaction is particularly relevant for the IL because deletion of one copy of *Sox2* diminishes tumorigenesis in *p27*^*−/−*^ animals (15).

The cellular specificity of this interaction may be explained by the restricted pattern of expression of SOX2 in pituitary endocrine cells; melanotrophs are the only endocrine cell type to express SOX2 (19). However, SOX2 is also expressed in adult pituitary SCs (2, 20, 21). Interestingly, in *p27*^*−/−*^ mice, the thickness of the SOX2^+ve^ SC layer is increased (15). Therefore, while the importance of the SOX2-P27 interaction for IL tumorigenesis is apparent, the modalities of this interaction and the roles of SOX2 in SCs and melanotrophs are unknown.

In this study, we have developed complementary genetic approaches to characterize the SOX2-P27 interaction and understand how *p27*^*−/−*^ IL tumorigenesis develops. As *p27*^*−/−*^ ILs become hyperplastic, melanotrophs tend to lose differentiated features and display increased levels of SOX2 expression in parallel with SCs. To determine the cellular origin of the tumors, and explore SOX2 function, we have performed lineage tracing experiments and deleted the gene separately in melanotrophs and SCs. This allows us to demonstrate that SOX2 is required in melanotrophs, and also, independently, in SCs for tumorigenesis. Deletion of the *Sox2* regulatory region 2 (*Srr2*), the target of P27 repressive action, further supports the protumorigenic role of SOX2 in SCs. Finally, single cell transcriptomic analyses reveal that activation of a SOX2-dependent MAPK pathway in SCs is important for tumorigenesis. In conclusion, our study shows that, following loss of *p27*, SOX2 orchestrates tumorigenesis both cell autonomously and noncell autonomously, uncovering an unexpected role for SCs in IL tumorigenesis. This suggests that SCs, or the relevant signaling molecules, represent good antitumoral targets, in addition to tumor cells themselves.

## Results

### Characterization of Intermediate Lobe Tumors in *p27*^*−/−*^ Pituitaries.

**Fig. 1.**
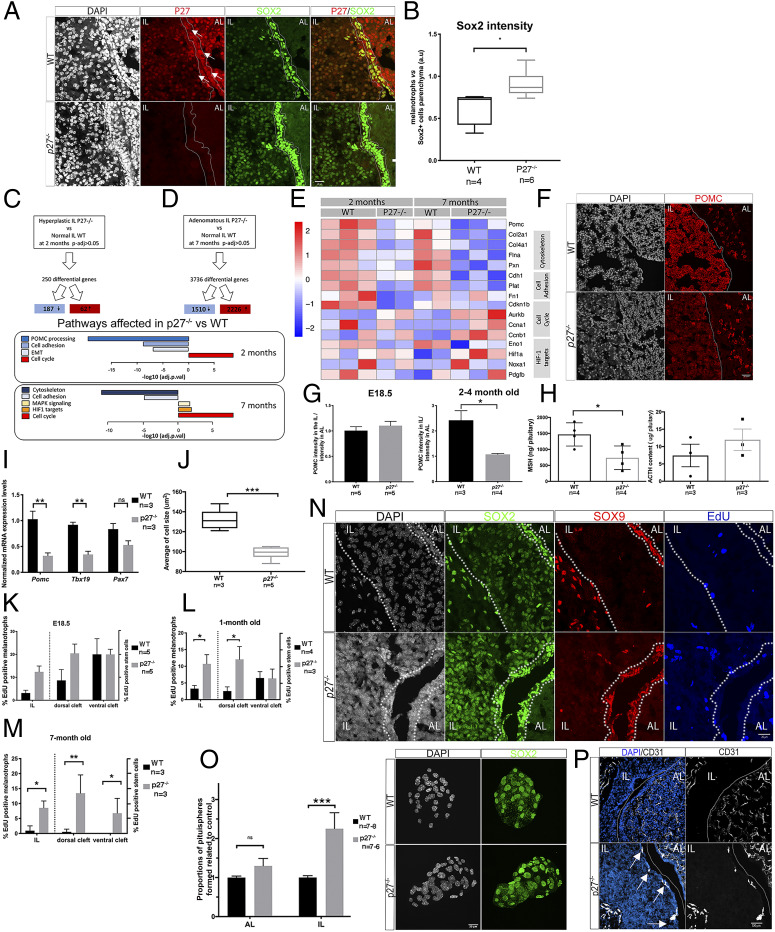
The expression of stem cell and differentiation markers is affected in *p27*^−/−^ IL. (*A*) P27 and SOX2 immunofluorescence in adult pituitaries. In *p27*^−/−^ IL, SOX2 staining is more intense. (*B*) Quantification of SOX2 expression levels in *p27*^−/−^ versus wild type (WT) after immunofluorescence. The ratio of staining intensity between SOX2^+^melanotroph/SOX2^+^ SC in AL parenchyma is represented. In WT, SOX2 staining in melanotrophs is less intense than in AL SCs (0.63 ± 0.20) while in *p27*^−/−^ it becomes similar (0.91 ± 0.16); (*P = 0.019, *n* = 4–6 in each group). (*C* and *D*) Bulk RNAseq selected pathway analysis in 2-mo-old IL (*p27*^−/−^ *n* = 2 vs. WT *n* = 3, adjusted *P* value <0.05) (*C*), and in 7-mo-old hyperplastic IL (*p27*^−/−^ *n* = 4 vs. WT *n* = 2) (*D*). Blue tones show down-regulated pathways while red-yellow represent up-regulation. (*E*) Heatmap of representative genes expression from pathways shown in *C* and *D*. (*F*) POMC immunofluorescence in adult pituitaries. (*G*) Quantification of POMC intensity in melanotrophs in relation to corticotrophs at E18.5 in WT (1.01 ± 0.17, *n* = 5) and *p27*^−/−^ (1.10 ± 0.19, *n* = 5) and in 2- to 4-mo-old animals. Levels are significantly reduced in mutants (WT: 2.42 ± 0.76, *n* = 3; *p27*^−/−^: 1.1 ± 0.07, *n* = 4, *P = 0.0286). (*H*) Radioimmunoassays for MSH and ACTH. *p27*^−/−^ pituitaries contain less MSH (*P = 0.0306, *n* = 4 in each group) (*Left* graph); ACTH levels are not affected (*Right *graph). (*I*) RT-qPCR in 2- to 3-mo-old *p27*^−/−^ and WT. *Pomc* (WT: 1.03 ± 0.27, *n *= 3; *p27*^−/−^: 0.32 ± 0.09, *n* = 3,**P = 0.0013) and *Tbx19* levels (WT: 0.94 ± 0.08, *n* = 3; *p27*^−/−^: 0.30 ± 0.10, *n* = 3, **P = 0.0071) were reduced in mutants. (*J*) Average cell size of melanotrophs. *p27*^−/−^ melanotrophs (99 ± 5.6, *n* = 5) are smaller compared to WT (132 ± 10, *n* = 3, ***P = 0.0007). (*K*–*M*) Analysis of cell proliferation. EdU incorporation was quantified in melanotrophs (EdU;SOX2;PAX7 triple positive/SOX2;PAX7 double positive in *L* or EdU;SOX2 double positive; SOX9 negative/SOX2 positive;SOX9 negative positive cells in *M* and *N*) and SCs (EdU;SOX2 double positive; PAX7 negative/SOX2 positive;PAX7 negative cells in *L* or EdU;SOX2;SOX9 triple positive/SOX2;SOX9 double positive in *M* and *N*). SCs were distinguished as ventral (flanking AL) or dorsal (flanking IL). In 1-mo-old mice (*M*), cell proliferation increases in *p27*^−/−^ melanotrophs and dorsal SCs (*n* = 3–4 mice/genotype, melanotrophs *P = 0.0235, dorsal cleft *P = 0.0419). In 7-mo-old mice, (*N*) cell proliferation increases in melanotrophs and both dorsal and ventral SCs (*n* = 3 mice/genotype, melanotrophs *P = 0.0167, dorsal cleft **P = 0.0085, ventral cleft *P = 0.0229). (*N*) SOX2, SOX9, and EdU triple staining in 1-mo-old pituitaries. (*O*) Immunofluorescence for SOX2 on IL pituispheres from 2- to 3-mo-old animals. The proportion of pituispheres is increased in mutant IL (***P < 0.0001, *n* = 6–8 in each group). (*P*) Immunofluorescence for CD31 in 1-mo-old pituitaries. *p27*^−/−^ ILs show ectopic blood vessels (arrows). ns, nonsignificant.

We first examined P27 by immunofluorescence and observed widespread nuclear expression throughout the anterior pituitary with varying levels of expression (Fig. 1*A*), in agreement with fluctuations of P27 according to the cell cycle. Anterior endocrine and stem cells proliferate rarely (20, 22) and P27 levels are relatively high in these. However, even though IL melanotrophs have been shown to be quiescent (6), P27 immunostaining is much weaker compared with the AL and SCs. We then examined SOX2, which is expressed both in SCs (18), and, at lower levels, in melanotrophs (19) (Fig. 1*A*). Coexpression of SOX2 and its repressor P27 probably reflects complex regulatory mechanisms controlling *Sox2* expression and/or availability of corepressors recruited by P27 mechanisms.

In *p27*^*−/−*^ pituitaries, the SC layer appears thicker in mutants (15). In agreement with a repressive action of P27 on *Sox2* expression (15), SOX2/*Sox2* levels are significantly up-regulated both in SCs and melanotrophs in *p27*^*−/−*^ pituitaries (Fig. 1 *A* and *B* and *SI Appendix*, Fig. S1 *A* and *B*).

To further characterize initiation and development of the tumors in *p27*^*−/−*^ mice, we performed transcriptomic analyses from dissected ILs (Fig. 1 *C*–*E* and *SI Appendix*, Fig. S1*C*). Before tumorigenesis, at 2 mo of age, we detected 250 differentially expressed genes (DEGs) between *p27*^*−/−*^ and control samples. In 7-mo-old animals, when hyperplasia is very clear, there were 3,736 DEGs (Dataset S1). We then performed a pathway analysis (Dataset S1). At both stages, up-regulation of cell cycle markers was observed, in agreement with the CDKI function of P27. We also observed down-regulation of genes associated with the cytoskeleton and cell adhesion which may reflect disruption of the direct interaction between P27 and RhoA (23). Furthermore, down-regulation of POMC and genes associated with its processing suggests an alteration of melanotroph function and/or identity in mutants.

We further investigated whether melanotroph function and/or identity have been altered, something that has not been reported previously (9–11). We observed a significant down-regulation of POMC processing in 2-mo-old mutants, while expression of *Pax7* and of the POMC-lineage marker *Tnxb* (24) are reduced in 7-mo-old mutants (Dataset S1). We then quantified POMC/*Pomc* expression levels, and detected a down-regulation exclusively in *p27*^*−/−*^ melanotrophs, postnatally, but prior to tumorigenesis (Fig. 1 *F*–*H*), while corticotrophs appear unaffected. In agreement with these data, radioimmunoassays show that levels of MSH are reduced while ACTH is unaffected in *p27*^*−/−*^ pituitaries (Fig. 1*H*). This shows that the anterior pituitary is affected by loss of *p27*. TBX19 is a direct upstream regulator required for *Pomc* expression (25). In agreement with a reduction in *Pomc* levels, *Tbx19* levels are significantly reduced in the IL, while *Pax7* levels (5) also appear reduced, although not to a statistically significant degree in 2- to 3-mo-old animals (Fig. 1*I*). Moreover, *p27*^*−/−*^ melanotrophs are smaller than those in wild-type (WT) pituitaries (Fig. 1*J*). Altogether, these results suggest that melanotroph identity and/or postnatal maturation are altered in *p27*^*−/−*^ mutants, and this occurs before tumors develop.

We then examined cell proliferation by quantifying incorporation of 5-ethynyl-2′-deoxyuridine (EdU) in the IL, dorsal (flanking IL), and ventral SC layer (flanking the AL) (Fig. 1 *K*–*N*). In E18.5 embryos, proliferation tends to increase in both IL and its flanking SC layer in the mutants; however, this is not statistically significant (Fig. 1*K*). In contrast, in 1-mo-old mutant animals, we observed a significant increase in proliferation of both melanotrophs and adjacent SCs (Fig. 1 *L*–*N*). In 7-mo-old animals, increased proliferation was observed in all compartments of the mutants compared to controls (Fig. 1*M*), in agreement with the transcriptomic analysis results (Fig. 1 *E* and *F*). To further test the proliferative capacity of SCs, we performed pituisphere assays (20) separately from anterior and intermediate lobe cells and observed an increased sphere-forming ability from *p27*^*−/−*^ IL cells (Fig. 1*O*). Altogether these results suggest that, while both SC layers behave abnormally in *p27*^*−/−*^ pituitaries, the SCs in direct contact with melanotrophs are more affected, suggesting that there may be some noncell autonomous consequences to the loss of *p27*.

HIF-1 activation is associated with tumor angiogenesis and its targets are up-regulated in 7-mo-old *p27*^*−/−*^ IL transcriptomes (Fig. 1*E*). Moreover, *p27*^*−/−*^ IL tumors are hemorrhagic (9). We thus examined CD31 expression and observed development of ectopic blood vessels in juvenile *p27*^*−/−*^ ILs, coincident with the abnormal increase in rates of cell proliferation (Fig. 1*P*).

Furthermore, *p27*^*−/−*^ mice are affected by gigantism, which has been attributed to general loss of cell cycle repression (9–11); however, an additional explanation could be the significant increase in pituitary growth hormone (GH) content we observed (*SI Appendix*, Fig. S1*D*). While anterior lobe tumors are not observed in mice (12), in both rats and humans carrying mutations in *P27* GH adenomas are observed (26). It would be of interest to examine whether in mice somatotroph numbers are affected.

In conclusion, *p27* deletion results in increased cell proliferation and ectopic vascularization in the IL. While P27 starts to be expressed in differentiating embryonic endocrine cells (13), defects consecutive to its loss appear to only become significant postnatally, both in melanotrophs and SCs. In addition, melanotroph identity and/or maturation appears affected in mutants.

### Characterization of the SOX2-P27 Interaction in *p27*^*−/−*^ Mutants.

**Fig. 2.**
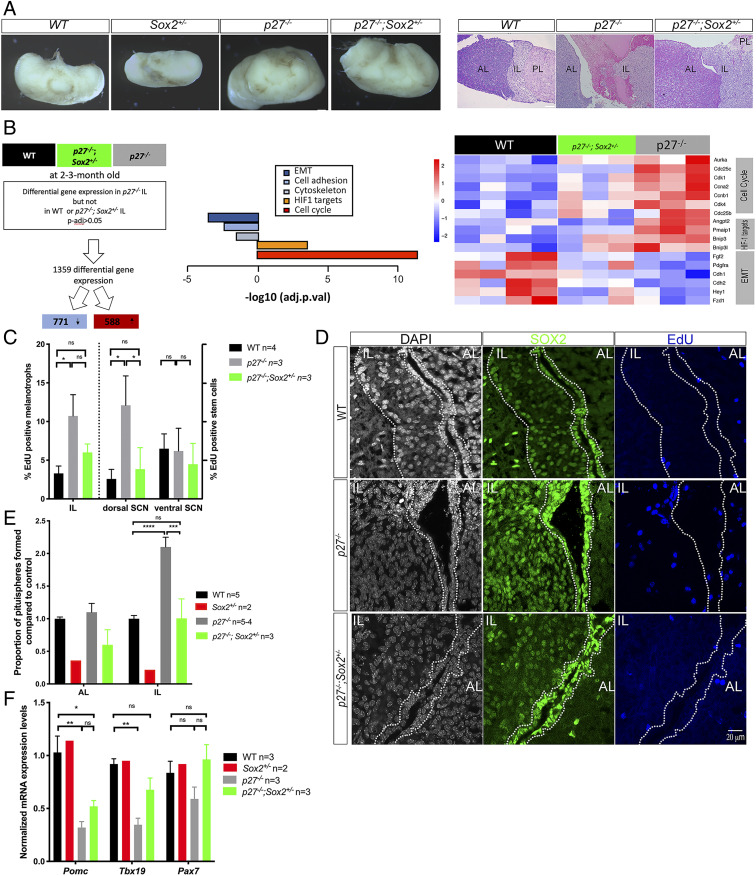
Deletion of one copy of *Sox2* in *p27*^*−/−*^ animals improves survival and results in reduction of cell proliferation and impairment of tumorigenesis. (*A*) Brightfield pictures taken at the same magnification of WT, *Sox2*^*+/−*^*, p27*^*−/−*^ , and *p27*^*−/−*^*; Sox2*^*+/−*^ pituitaries (*Left*). Histological analysis of 10- to 12-mo-old WT, *p27*^*−/−*^, and *p27*^*−/−*^*; Sox2*^*+/−*^ pituitaries (*Right*). In *p27*^*−/−*^*; Sox2*^*+/−*^ IL tumorigenesis is impaired, but hyperplasia is observed. (*B*) Comparison modalities for RNAseq analysis of wild-type, *p27*^*−/−*^, and *p27*^*−/−*^*; Sox2*^*+/−*^ 2- to 3-mo-old IL samples (WT *n* = 4, *p27*^*−/−*^ *n* = 3, *p27*^*−/−*^*; Sox2*^*+/−*^ *n* = 3, adjusted *P* value <0.05) (*Left*). Selected enriched pathways are represented (*Center*) and heatmap of representative gene expression from affected pathways (*Right*). (*C*) Analysis of cell proliferation. EdU incorporation was quantified in melanotrophs (EdU; SOX2 double positive; SOX9 negative/SOX2 positive;SOX9 negative cells) and SCs (EdU; SOX2; SOX9 triple positive/SOX2; SOX9 double positive cells). SCs were further distinguished as ventral or dorsal, respectively, as flanking the anterior or intermediate lobe. Deletion of one copy of *Sox2* results in a reduction of cell proliferation compared to *p27*^*−/−*^ samples in dorsal cleft SCs (*n* = 3 to 4 mice/genotype, *P = 0.0208), melanotrophs. (*D*) SOX2 and EdU double staining in 2- to 3-mo-old WT, *p27*^*−/−*^, and *p27*^*−/−*^*; Sox2*^*+/−*^ animals. There is a clear reduction in SOX2 levels in *p27*^*−/−*^*; Sox2*^*+/−*^ ILs. Cleft and IL are outlined. (*E*) Proportion of pituispheres obtained from AL and IL WT, *Sox2*^+/−^, *p27*^*−/−*^, and *p27*^*−/−*^*; Sox2*^*+/−*^ 2- to 3-mo-old animals. There is a reduction in the proportion of pituispheres formed from *p27*^*−/−*^*; Sox2*^*+/−*^ ILs compared to *p27*^*−/−*^ samples. WT (1 ± 0.11, *n* = 5), *Sox2*^*+/−*^ (0.2 ± 0.08, *n* = 2), *p27*^*−/−*^ (2.09 ± 0.31, *n* = 4), and *p27*^*−/−*^*; Sox2*^*+/−*^ (1 ± 0.52, *n* = 3), ****P < 0.0001 and ***P = 0.0002; the reduction in *Sox2*^*+/−*^ samples in agreement with the slightly hypomorphic pituitaries observed in *Sox2* heterozygous mice (25). (*F*) Quantitative analysis of melanotroph marker expression levels by RT-qPCR in 2- to 3-mo old WT, *Sox2*^*+/−*^, *p27*^*−/−*^, and *p27*^*−/−*^ *; Sox2*^*+/−*^ IL. In *p27*^*−/−*^*; Sox2*^*+/−*^ animals, *Tbx19* expression levels are restored to WT levels. *Pomc*; WT (*n* = 3) vs. *p27*^*−/−*^*; Sox2*^*+/−*^ (*n* = 3), *P = 0.03 and WT vs. *p27*^*−/−*^ (*n* = 3), **P = 0.0022, *Tbx19*; WT vs. *p27*^*−/−*^*; Sox2*^*+/−*^ (*n* = 3, *P* = nonsignificant [ns]) and WT vs. *p27*^*−/−*^ (*n* = 3, *P = 0.0169).

Deletion of one copy of *Sox2* is sufficient to dramatically reduce occurrence of IL tumors (Fig. 2*A* and *SI Appendix*, Fig. S2*B*) (15); however, hyperplasia is still observed in *p27*^*−/−*^*; Sox2*^*+/−*^ IL (Fig. 2*A*). We further characterized the SOX2-P27 interaction by demonstrating that survival of *p27*^*−/−*^ mutants (12.5 mo, *n* = 28) was significantly improved by *Sox2* haploinsufficiency (17.1 mo, *n* = 10) (*SI Appendix*, Fig. S2*A*). However, in agreement with cell-type specificity for the SOX2-P27 interaction, while pituitary and duodenum tumors were sensitive to *Sox2* dosage, lung tumor development was unaffected (*SI Appendix*, Fig. S2*B*).

We then performed comparisons of the transcriptomes of wild type, *p27*^*−/−*^, and *p27*^*−/−*^*; Sox2*^*+/−*^ IL (Fig. 2*B* and *SI Appendix*, Fig. S2*C*). To pinpoint genes underlying IL tumor impairment in compound mutants, and conversely those associated with higher levels of SOX2 and tumorigenesis, we focused our analyses on those that were exclusively differentially expressed in *p27*^*−/−*^, and therefore restored to control levels in *p27*^*−/−*^*; Sox2*^*+/−*^ samples. This analysis was thus conducted differently than the one presented in Fig. 1 *C* and *D*, where we simply compared *p27*^*−/−*^ vs. wild type. When compared, the two lists of DEGs comprise both similarities and differences (Figs. 1*E* and 2*B* and Datasets S1 and S2). However, when we performed a pathway analysis, we obtained a list of pathways comparable in our two analyses (Figs. 1 *C* and *D* and 2*B* and Datasets S1 and S2). This implies that in the IL, the different processes affected by *P27* loss are restored to normal levels upon deletion of one copy of *Sox2*. This thus suggests that SOX2 is not required for a specific aspect of tumorigenesis in *p27*^*−/−*^ mice, but rather that it has a broad and therefore probably early role in tumor formation. Reduced *Sox2* dosage is associated with a decrease in proliferation and HIF1-target gene expression, in agreement with less angiogenesis linked with reduced overproliferation in *p27*^*−/−*^*; Sox2*^*+/−*^ mutants. We also observed an effect on genes involved in the cytoskeleton, suggesting that reduction of *Sox2* allows restoration of a cellular phenotype resembling that of wild type. Alternatively, alteration of cellular shape and contacts may be linked to variation in rates of cell proliferation.

We then analyzed SOX2 expression, cell proliferation, and pituisphere formation in *p27*^*−/−*^*; Sox2*^*+/−*^ pituitaries (Fig. 2 *C*–*E*). There was a reduction in SOX2/*Sox2* levels in *p27*^*−/−*^*; Sox2*^*+/−*^ IL, both in melanotrophs and stem cells (Fig. 2*D* and *SI Appendix*, Figs. S2*D*, S3*E*, and S4*J*). Moreover EdU incorporation in compound mutants, both in melanotrophs and SCs was not significantly different from controls, in agreement with impaired tumorigenesis (Fig. 2*C*). A similar effect was observed in pituisphere assays (Fig. 2*E*). We moreover observed a reduction in pituisphere formation efficiency in *Sox2*^*+/−*^ samples, suggesting a reduced proliferative capacity (Fig. 2*E*). This is in agreement with the slightly hypomorphic pituitaries observed in *Sox2* heterozygous mice (27) and with SOX2 being required for proliferation of pituitary progenitors in the embryo (19, 28).

Alteration of *p27*^*−/−*^ melanotroph identity (Fig. 1*I*) also appears to depend on SOX2 dosage, because levels of *Tbx19* which are reduced in *p27*^*−/−*^ IL, are not significantly affected in *p27*^*−/−*^*; Sox2*^*+/−*^ compared to control IL (Fig. 2*F* and see Fig. 6*J* below). However, levels of *Pomc* were still significantly reduced in *p27*^*−/−*^*; Sox2*^*+/−*^ IL compared to control.

### Sox2 Regulatory Region 2 (Srr2) Mediates P27 Effect on Sox2 Expression In Vivo.

**Fig. 3.**
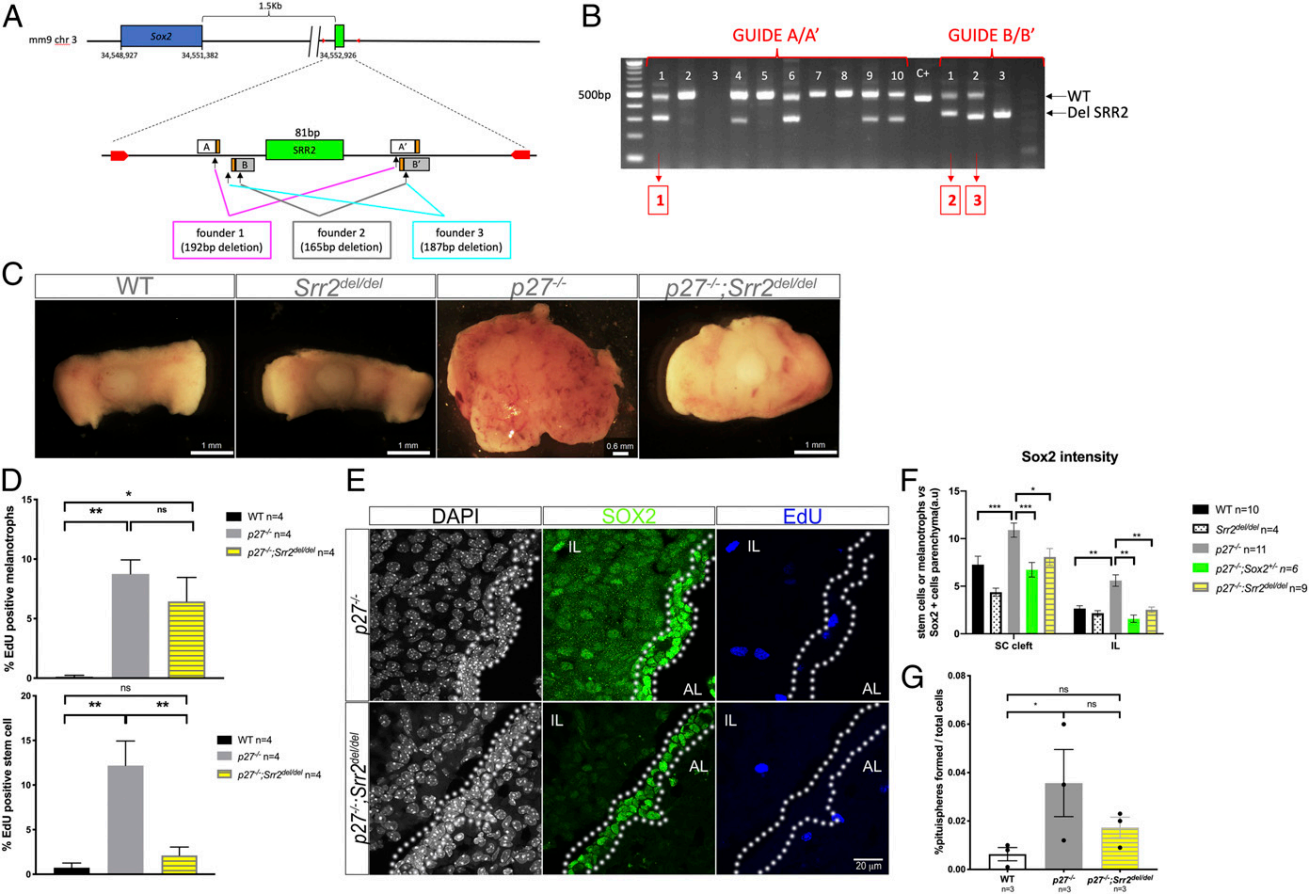
Deletion of *Srr2* in *p27*^*−/−*^ animals results in reduction of cell proliferation in SCs and impairment of tumorigenesis. (*A*) Schematic representation of the *Sox2* murine locus and *Srr2* deletions. Two pairs of sgRNA (pairs 1 and 2, protospacer adjacent motif sequence in orange) were designed. The primers used to genotype *Srr2*-deleted animals are marked by red arrows. Three founders deleted for *Srr2* were used to generate stable lines (founders 1 through 3); the size of the deletion is indicated for each line. (*B*) Gel electrophoresis showing mosaic loss of *Srr2* in founder mice. The number above each lane corresponds to a individual founder. Boxed numbers below indicate strain derived from that particular founder. (*C*) Brightfield pictures of WT, *Srr2*^*del/del*^, *p27*^*−/−*^, and *p27*^*−/−*^*; Srr2*^*del/del*^ pituitaries. Deletion of two copies of *Sox2* Srr2 on a *p27*^*−/−*^ background is sufficient to delay tumorigenesis. (*D*) Analysis of cell proliferation. EdU incorporation was quantified in melanotrophs (EdU; SOX2 double positive; SOX9 negative/SOX2 positive; SOX9 negative cells) and SCs (EdU; SOX2; SOX9 triple positive/SOX2; SOX9 double positive cells) in 7-mo-old WT, *p27*^*−/−*^, and *p27*^*−/−*^*; Srr2*^*del/del*^ animals. In *p27*^*−/−*^*; Srr2*^*del/del*^ animals, there is a reduction of proliferation in stem cells compared to *p27*^*−/−*^ (*p27*^*−/−*^ (12.33 ± 5.37, *n* = 4) vs. *p27*^*−/−*^*; Srr2*^*del/del*^ (2.11 ± 1.88, *n* = 4), **P = 0.0061), but not in melanotrophs (*p27*^*−/−*^ [8.75 ± 2.36] vs. *p27*^*−/−*^*; Srr2*^*del/del*^ [6.45 ± 4.01], *P* = ns). (*E*) SOX2 and EdU double staining in 7-mo-old *p27*^*−/−*^ and *p27*^*−/−*^*; Srr2*^*del/del*^ animals. SOX2 expression appears decreased *in p27*^*−/−*^*; Srr2*^*del/del*^ IL. Cleft is outlined. (*F*) Quantification of SOX2 expression levels in WT, *Srr2del/del, p27*^−/−^, *p27*^−/−^*;Sox2*^+/−^, *p27*^−/−^*;Srr2del/del*. Levels of expression were quantified after immunofluorescence in SCs and melanotrophs. In *p27*^−/−^*;Srr2del/del*, SOX2 staining is less intense than in *p27*^−/−^ (*P = 0.017 SCs and **P = 0.0078 in melanotrophs, *n* = 11 to 4 in each group). In contrast SOX2 staining is more intense in *p27*^−/−^ SC vs. WT (***P = 0.0007) and *p27*^−/−^*;Sox2*^+/−^ (***P = 0.0009). (*G*) Proportion of pituispheres obtained from IL in WT, *p27*^*−/−*^, and *p27*^*−/−*^*; Srr2*^*del/del*^. The percentage of spheres formed from *p27*^*−/−*^*; Srr2*^*del/del*^ (0.02 ± 0.007, *n* = 3, *P* = ns) is not significantly higher than WT (0.006 ± 0.005, *n* = 3), in contrast with *p27*^*−/−*^ samples (0.04 ± 0.02, *n* = 3, *P = 0.0344), demonstrating that loss of *Srr2* has an impact on sphere-forming efficiency in mutants. ns, nonsignificant.

Transcriptomic studies and proliferation and marker analyses all indicate that reduction of SOX2 dosage compromises most aspects of tumorigenesis in *p27*^*−/−*^ animals, implying an essential role for SOX2 in *p27*^*−/−*^ melanotrophs and adjacent SCs for tumor development. To test whether the SOX2-P27 interaction relies solely on *Srr2* (15), we generated mice deleted for this region (Fig. 3 *A* and *B*). *Srr2*^*del/del*^ mutants were viable (*SI Appendix*, Fig. S3*A*) and pituitary morphology was normal (Fig. 3*C*). However, on a *p27*^*−/−*^ background, the *Srr2* deletion prevents tumorigenesis, while hyperplasia of IL is still evident, as observed in *p27*^*−/−*^*; Sox2*^*+/−*^ mutants (Fig. 3*C* and *SI Appendix*, Fig. S3*B*). In addition, the morphology of the retina, which is affected in absence of *p27* (11), is also improved in the *p27*^*−/−*^*; Srr2*^*del/del*^ mutants. In particular, and again similarly to *p27*^*−/−*^*; Sox2*^*+/−*^ mutants, the inner and outer layers have a reduced thickness and better organization compared to *p27*^*−/−*^ retina (*SI Appendix*, Fig. S3*C*) (15). In the pituitary, levels of SOX2 are reduced in both melanotrophs and SCs in *p27*^*−/−*^*; Srr2*^*del/del*^ mutants compared to *p27*^*−/−*^ samples (Fig. 3 *E* and *F* and *SI Appendix*, Fig. S3*D*). Intriguingly, EdU incorporation analyses revealed a specific reduction of proliferation in SCs in compound mutants compared to *p27*^*−/−*^ samples (Fig. 3*D*). In agreement with this result, while cultures from *p27*^*−/−*^ IL give rise to significantly more pituispheres than controls, the efficiency of pituisphere formation from *p27*^*−/−*^*; Srr2*^*del/del*^ samples is reduced (Fig. 3*G*). These analyses strongly suggest that *Srr2* has an exclusive role in mediating the SOX2-P27 interaction (see model *SI Appendix*, Fig. S3*E*). Moreover, the consequences of its deletion are more obvious in SCs, suggesting that *Srr2* plays a more important role in this compartment. Furthermore, this implies that SCs may be critical for IL tumorigenesis in *p27*^*−/−*^ mutants.

### Requirement for SOX2 in *p27*^*−/−*^ Melanotrophs.

**Fig. 4.**
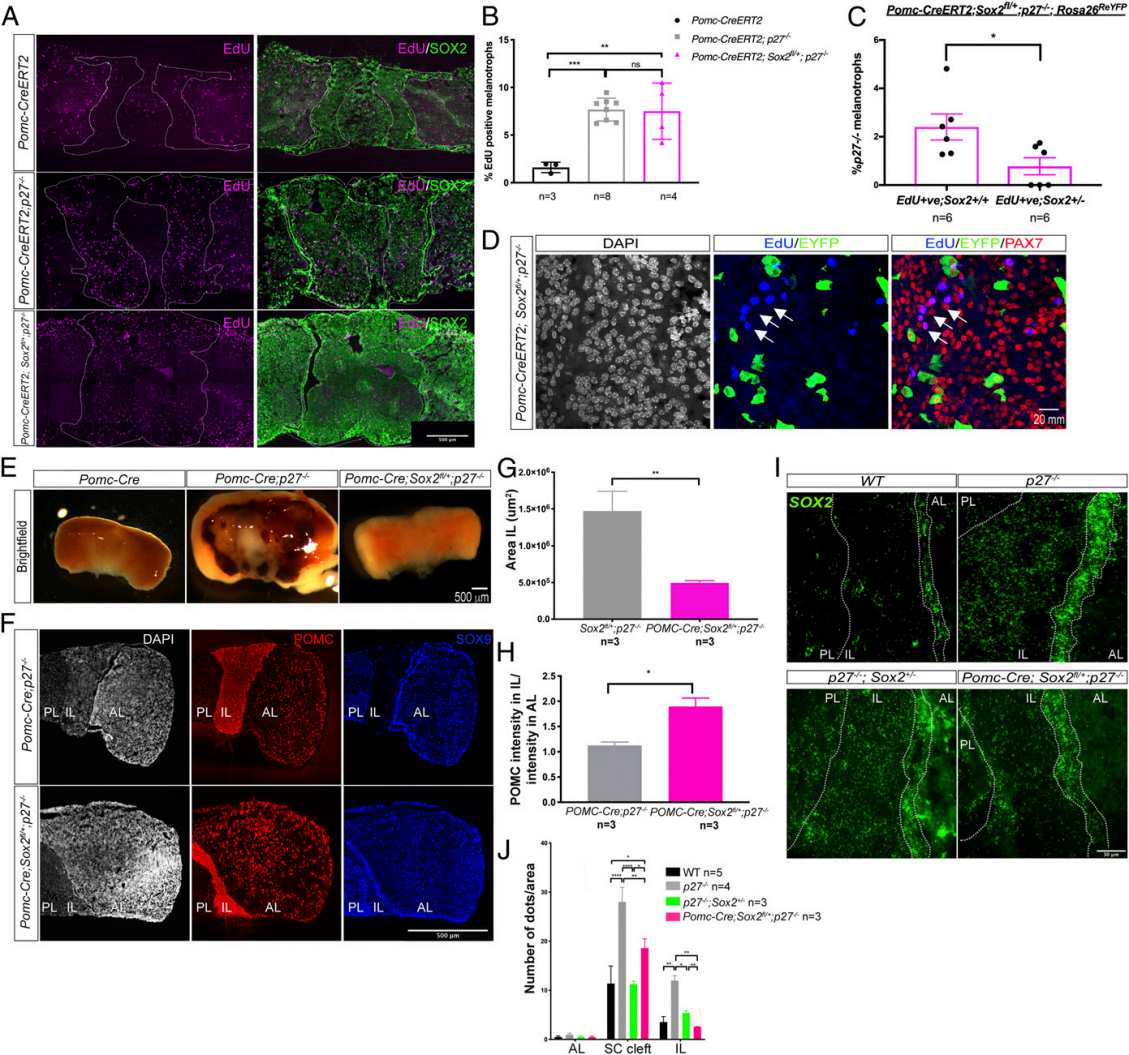
Deletion of one copy of *Sox2* in *p27*^*−/−*^ melanotrophs prevents IL tumorigenesis. (*A*) SOX2 and EdU double staining in 6- to 8-mo-old *Pomc-CreERT2*, *Pomc-CreERT2; p27*^*−/−*^, and *Pomc-CreERT2; Sox2*^*fl/+*^*; p27*^*−/−*^ pituitaries. (*B*) Analysis of cell proliferation. EdU incorporation was quantified in melanotrophs (EdU; SOX2 low; POMC triple positive/DAPI nuclei in IL). EdU levels are similarly elevated in both *p27*^*−/−*^ samples. *Pomc-CreERT2; Sox2*^*fl/+*^*; p27*^*−/−*^ (7.53 ± 2.92, *n* = 4, **P = 0.0023) and *Pomc-CreERT2; p27*^*−/−*^ (7.68 ± 1.20, *n* = 8, ***P = 0.0007) vs. *Pomc-CreERT2* (controls), (1.60 ± 0.55, *n* = 3). (*C* and *D*) EdU incorporation was quantified in *Sox2*^*+/+*^ melanotrophs (PAX7^+ve^; eYFP^−ve^) (2.40 ± 1.31, *n* = 6) and in *Sox2*^*+/−*^ melanotrophs (PAX7; eYFP double positive) (0.78 ± 0.86, *n* = 6) in 2.5- to 6-mo-old *Pomc-CreERT2; Sox2*^*fl/+*^*; p27*^*−/−*^*; Rosa26*^*ReYFP*^ mice, assuming that *Rosa26*^*ReYFP*^ recombination reflects *Sox2* heterozygous deletion. In *Sox2*^*+/−*^ melanotrophs EdU incorporation is significantly reduced compared to *Sox2*^*+/+*^ ones (*P = 0.0210), demonstrating that loss of one copy of *Sox2* results in reduced proliferation in *p27*^*−/−*^ melanotrophs. (*D*) EdU, eYFP, and PAX7 triple staining. Most EdU; PAX7 positive cells (arrows) do not express eYFP in *Pomc-CreERT2; Sox2*^*fl/+*^*; p27*^*−/−*^*; Rosa26*^*ReYFP*^ IL. (*E*) Brightfield pictures of *Pomc-Cre, Pomc-Cre; p27*^*−/−*^, and *Pomc-Cre; Sox2*^*fl/+*^*; p27*^*−/−*^ pituitaries. Deletion of one copy of *Sox2* using Pomc-Cre prevents IL tumorigenesis. (*F*) Double immunofluorescence for POMC and SOX9 in 6- to 10-mo-old animals, illustrating reduced IL size in *Pomc-Cre; Sox2*^*fl/+*^*; p27*^*−/−*^. (*G*) Measurement of IL area in *Pomc-Cre; p27*^*−/−*^ *(*1,472,054 ± 808,382, *n* = 3) and Pomc-Cre; Sox2^fl/+^; p27^−/−^ (495,612 ± 185,861, *n* = 3). IL area *in Pomc-Cre; Sox2*^*fl/+*^*; p27*^*−/−*^ pituitaries represents a third of IL area in *p27*^*−/−*^ mutants (**P = 0.0041). (*H*) Quantification of POMC intensity in melanotrophs in relation to corticotrophs in 6- to 10-mo-old *Pomc-Cre; p27*^*−/−*^ (1.13 ± 0.11, *n* = 3) vs. *Pomc-Cre; Sox2*^*fl/+*^*; p27*^*−/−*^ (1.89 ± 0.29, *n* = 3) animals, where levels are significantly increased, *P = 0.0127. (*I*) In situ hybridization for *Sox2* in 6.25- to 10-mo-old wild-type, *p27*^*−/−*^, *p27*^*−/−*^*; Sox2*^*+/−*^, and *Pomc-Cre; Sox2*^*fl/+*^*; p27*^*-/*−^ animals. (*J*) Quantification of *Sox2* levels following in situ hybridization. *Sox2* expression levels are reduced in both *p27*^*−/−*^*; Sox2*^*+/−*^ and *Pomc-Cre; Sox2*^*fl/+*^*; p27*^*-/*−^ melanotrophs and SCs compared to *p27*^*−/−*^. Quantification of *Sox2* levels in melanotrophs: *p27*^*−/−*^ (12.17 ± 2.15, *n* = 4), WT (3.5 ± 2.5, **P < 0.003), *p27*^*−/−*^*; Sox2*^*+/−*^ (5.4 ± 1, *P = 0.0276), *Pomc-Cre; Sox2*^*fl/+*^*; p27*^*−/*−^ (2.56 ± 0.1, **P = 0.0038). Quantification of *Sox2* levels in SCs: *p27*^*−/−*^ (28 ± 6, *n* = 4), WT (11.4 ± 8, *n* = 5, ****P < 0.0001), *p27*^*−/−*^*; Sox2*^*+/−*^ (11.3 ± 1.4, *n* = 3, ****P < 0.0001), *Pomc-Cre; Sox2*^*fl/+*^*; p27*^*−/*−^ (19 ± 3, *n* = 3, **P = 0.0039). IL is outlined in *A*. IL and stem cell cleft are outlined in *I*. ns, nonsignificant.

Conditional deletion of *p27* in melanotrophs results in formation of tumors; therefore, transformation is cell autonomous in this context (29). To better characterize the role of SOX2 for tumorigenesis, we deleted one copy of the gene exclusively in melanotrophs in *p27*^*−/−*^ mutants. We initially used *Pomc-CreERT2* which displays a mosaic pattern of recombination in the IL (*SI Appendix*, Fig. S4*A*). To look at heterozygosity for *Sox2*, we induced Cre activity in 4-wk-old *Pomc-CreERT2; Sox2*^*fl/+*^*; p27*^*−/−*^ animals and controls, which were then examined 6 to 8 mo later. Tumor development was unaffected by the mosaic deletion of one copy of *Sox2* in *p27*^*−/−*^ melanotrophs (Fig. 4*A*). In agreement with this observation, proliferation was unaffected by *Sox2* reduction, as we observed a similar increase in EdU incorporation in *Pomc-CreERT2; Sox2*^*fl/+*^*; p27*^*−/−*^ and *Pomc-CreERT2; p27*^*−/−*^ IL compared to controls (Fig. 4 *A* and *B*). To analyze more directly the effect of the loss of one copy of *Sox2* on melanotrophs’ proliferative ability, we analyzed EdU incorporation in *Pomc-CreERT2; Sox2*^*fl/+*^*; p27*^*−/−*^*; Rosa26*^*ReYFP*^ samples. We compared the percentages of eYFP; PAX7; EdU triple positive and eYFP negative, PAX7; EdU double positive melanotrophs, assuming that *Rosa26*^*ReYFP*^ recombination also reflected *Sox2* heterozygous deletion. We observed a significant reduction of EdU incorporation in eYFP-positive vs. -negative cells (Fig. 4 *C* and *D*). Therefore, reduction of SOX2 dosage in *p27*^*−/−*^ melanotrophs diminishes their proliferative capacity.

We then hypothesized that a more efficient Cre-driver may block tumorigenesis; we therefore generated *Pomc-Cre; Sox2*^*fl/+*^*; p27*^*−/−*^animals. We showed previously that SOX2 is required for acquisition of melanotroph identity; however, homozygous deletion of the gene once melanotrophs have up-regulated PAX7 does not have an apparent effect on cell fate acquisition (19). There were no IL tumors or signs of hyperplasia in any 4- to 7-mo-old *Pomc-Cre; Sox2*^*fl/+*^*; p27*^*−/−*^ animals (Fig. 4 *E*–*G* and *SI Appendix*, Fig. S4*B*). Moreover POMC staining intensity was increased in *Pomc-Cre; Sox2*^*fl/+*^*; p27*^*−/−*^ melanotrophs compared to *p27*^*−/−*^ samples (Fig. 4*H*), indicating restoration of a normal melanotroph phenotype. This demonstrates that SOX2 is required in melanotrophs for *p27* deletion to result in tumorigenesis. Strikingly, the morphology of IL appears completely normal in *Pomc-Cre; Sox2*^*fl/+*^*; p27*^*−/−*^ (Fig. 4 *E* and *F*) compared to *p27*^*−/−*^*; Sox2*^*+/−*^ IL where hyperplasia is clear (Fig. 2*A*). This difference in phenotype is unexpected, because in both mutants the genotype of melanotrophs is the same: only one allele of *Sox2* is active while *p27* is deleted. However, different regulatory mechanisms may affect the levels of expression of the remaining copy of *Sox2* in *Pomc-Cre; Sox2*^*fl/+*^*; p27*^*−/−*^ vs. *p27*^*−/−*^*; Sox2*^*+/−*^ melanotrophs. We thus quantified *Sox2* levels and observed an up-regulation in *p27*^*−/−*^ , and to a lesser extent in *p27*^*−/−*^*; Sox2*^*+/−*^ samples (Fig. 4 *I* and *J*). In agreement with the absence of hyperplasia, we observed a further reduction in *Sox2* expression in *Pomc-Cre; Sox2*^*fl/+*^*; p27*^*−/−*^ vs. *p27*^*−/−*^*; Sox2*^*+/−*^ samples, suggesting that regulation of *Sox2* is indeed modulated according to the spatiotemporal pattern of its deletion. Furthermore we also observed, albeit to a lesser extent, reduction of *Sox2* expression in *Pomc-Cre; Sox2*^*fl/+*^*; p27*^*−/−*^ SCs.

In conclusion, while cell transformation in *p27*^*−/−*^ melanotrophs is known to be cell autonomous (29), we demonstrate here that SOX2 is required in these cells for tumor development in *p27*^*−/−*^ mutants. Moreover, reduction of *Sox2* expression in SCs in *Pomc-Cre; Sox2*^*fl/+*^*; p27*^*−/−*^ compared to *p27*^*−/−*^ suggests a noncell autonomous role and implies an interaction between melanotrophs and SCs.

### SOX2 Is Required Independently in SCs for Induction of Melanotroph Tumors in *p27*^*−/−*^ Animals.

**Fig. 5.**
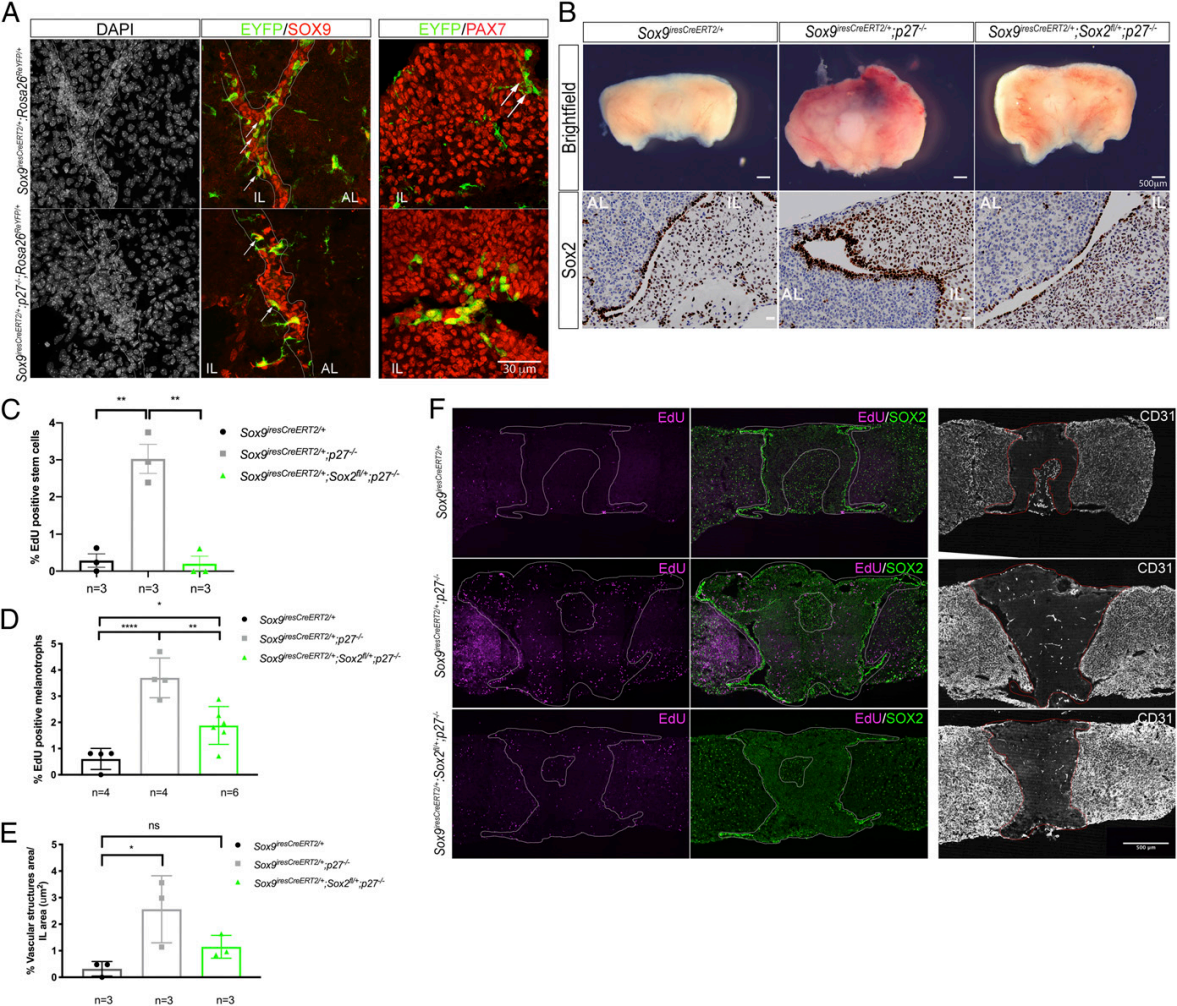
SOX2 is required in SCs for tumorigenesis in *p27*^*−/−*^ IL. (*A*) eYFP, SOX9 (*Middle*), and eYFP, PAX7 (*Right*) double stainings in 7- to 12-mo-old *Sox9*^*iresCreERT2/+*^*; Rosa26*^*ReYFP/+*^ and *Sox9*^*iresCreERT2/+*^*; p27*^*−/−*^*; Rosa26*^*ReYFP/+*^ animals. eYFP; SOX9 double positive cells are localized in the epithelium lining the cleft in both genotypes (arrows). Very few eYFP^+^, SOX9^−^, and PAX7^−^ cells are present in *Sox9*^*iresCreERT2/+*^*; Rosa26*^*ReYFP/+*^ animals (arrows). In *Sox9*^*iresCreERT2/+*^*; p27*^*−/−*^ *Rosa26*^*ReYFP/+*^ pituitaries some rare eYFP; PAX7 double-positive cells are present, demonstrating low levels of differentiation into melanotroph (*Left*). (*B*) Brightfield pictures (*Upper*) and SOX2 immunohistochemistry (*Bottom*) on sections of Sox9^iresCreERT2/+^; Rosa26^ReYFP/+^, Sox9^iresCreERT2/+^; p27^−/−^, and Sox9^iresCreERT2/+^; Sox2^fl/+^; p27^−/−^ pituitaries. In Sox9^iresCreERT2/+^; Sox2^fl/+^; p27^−/−^ pituitaries tumorigenesis is impaired (1-y-old 14% tumor incidence, *n* = 7) compared to Sox9^iresCreERT2/+^; p27^−/−^ (1-y-old, *n* = 5, 100% tumor incidence). The thickness of the SOX2 positive SC epithelium resembles that of control, demonstrating that reduction of Sox2 dosage in SCs impairs p27^−/−^ IL tumorigenesis. (*C*, *D*, and *F*) Analysis of cell proliferation. EdU incorporation was quantified in SCs and melanotrophs in 7- to 12-mo-old *Sox9*^*iresCreERT2/+*^, *Sox9*^*iresCreERT2/+*^*; p27*^*−/−*^, and *Sox9*^*iresCreERT2/+*^*; Sox2*^*fl/+*^*; p27*^*−/−*^ IL. (*C*) In SCs (EdU; SOX2; SOX9 triple positive/SOX2; SOX9 double-positive cells), there is a significant reduction of proliferation in *Sox9*^*iresCreERT2/+*^*; Sox2*^*fl/+*^*; p27*^*−/−*^ samples (0.20 ± 0.35, *n* = 3, **P = 0.0011) compared to *Sox9*^*iresCreERT2/+*^*; p27*^*−/−*^ ones (3 ± 0.68, *n* = 3). In fact, proliferation in *Sox9*^*iresCreERT2/+*^*; Sox2*^*fl/+*^*; p27*^*−/−*^ SC is similar to *Sox9*^*iresCreERT2/+*^ controls (0.28 ± 0.32, *n* = 3, *P* = ns). (*D*) In melanotrophs (POMC positive cells in IL/DAPI positive nuclei), there is a reduction of cell proliferation in *Sox9*^*iresCreERT2/+*^*;Sox2*^*fl/+*^*;p27*^−/−^ samples (1.88 ± 0.72, *n* = 6, **P = 0.0033) compared to *Sox9*^*iresCreERT2/+*^*;p27*^−/−^ (3.7 ± 0.76, *n* = 4); again proliferation in *Sox9*^*iresCreERT2/+*^*;Sox2*^*fl/+*^*;p27*^−/−^ is similar to *Sox9*^*iresCreERT2/+*^ controls (0.60 ± 0.4, *n* = 4, *P = 0.03) while proliferation is increased in *Sox9*^*iresCreERT2/+*^*;p27*^−/−^ vs *Sox9*^*iresCreERT2/+*^ controls (****P = 0.0001). (*E*) Quantification of the vascular structures ectopic development in 7 to 12 mo old. *Sox9*^*iresCreERT2/+*^, *Sox9*^*iresCreERT2/+*^*; p27*^*−/−*^, and *Sox9*^*iresCreERT2/+*^*; Sox2*^*fl/+*^*; p27*^*−/−*^ animals. Deletion of one copy of *Sox2* in SC leads to a reduction in the development of ectopic blood vessels in IL. IL vascular structures area: *Sox9*^*iresCreERT2/+*^ (0.32 ± 0.28, *n* = 3), *Sox9*^*iresCreERT2/+*^*; p27*^*−/−*^ (2.56 ± 1.26, *n* = 3, (*P < 0.03), and *Sox9*^*iresCreERT2/+*^*; Sox2*^*fl/+*^*; p27*^*−/−*^ (1.15 ± 0.43, *n* = 3). (*F*) EdU, SOX2 double staining in 7- to 12-mo-old *Sox9*^*iresCreERT2/+*^, *Sox9*^*iresCreERT2/+*^*; p27*^*−/−*^, and *Sox9*^*iresCreERT2/+*^*; Sox2*^*fl/+*^*; p27*^*−/−*^ pituitary glands. *Right*, immunofluorescence for CD31 showing ectopic blood vessel formation reduction in *Sox9*^*iresCreERT2/+*^*; Sox2*^*fl/+*^*; p27*^*−/−*^ sample. SC layer is outlined in *A*, IL in *F*. ns, nonsignificant.

Because deletion of *p27* induces up-regulation of SOX2 in SCs, we asked whether the SCs flanking the IL directly give rise to tumor cells or induce tumorigenesis. Given that, in the context of the pituitary, SOX9 is expressed uniquely in the SCs (20), we first performed lineage tracing experiments using *Sox9*^*iresCreERT2*^ (30). Cre activity was induced in 4-wk-old *Sox9*^*iresCreERT2/+*^*; Rosa26*^*ReYFP/+*^ animals and pituitaries were harvested 6 to 11 mo later. In controls, the vast majority of eYFP-positive cells are SOX9-positive SCs (21) (Fig. 5*A*). In agreement with a low turnover of melanotrophs (6), very few eYFP-positive cells are present in the IL (Fig. 5*A*); these are negative for SOX9 and PAX7, where the latter indicates they are not melanotrophs. Their identity is unclear; they may originate from the pituitary SC layer or the posterior lobe where SOX9 is also expressed. In *p27*^−/−^ animals we also observed that most eYFP-positive cells are SOX9 positive SCs. However, in contrast with *Sox9*^*iresCreERT2/+*^*; Rosa26*^*ReYFP/+*^ controls, we observed some rare eYFP; PAX7-positive melanotrophs. These results clearly demonstrate that SCs are not at the origin of the melanotroph tumors in *p27*^−/−^ animals. However, tumorigenesis and/or lack of *p27* in SCs, drives commitment of rare SCs toward the melanotroph lineage.

To investigate the role of SOX2 in SCs during tumorigenesis, one allele of the gene was removed exclusively in SCs in *Sox9*^*iresCreERT2/+*^*; Sox2*^*fl/+*^*; p27*^*−/−*^ animals. Cre activity was induced in 4-wk-old animals and pituitaries harvested when the animals were 6 to 12 mo old. Strinkingly, only one among seven 12-mo-old *Sox9*^*iresCreERT2/+*^*; Sox2*^*fl/+*^*; p27*^*−/−*^ animals developed an IL tumor, while all *p27*^*−/−*^controls were affected (Fig. 5 *B*, *Upper* and *SI Appendix*, Fig. S5*A*). The IL is however consistently hyperplastic in *Sox9*^*iresCreERT2/+*^*; Sox2*^*fl/+*^*; p27*^*−/−*^ animals, similarly to *p27*^*−/−*^*; Sox2*^*+/−*^ mice. These results unequivocally demonstrate that SOX2 is required independently in SCs for tumors to develop in *p27*^*−/−*^ IL. The thickness of the SOX2-positive SC layer appeared reduced in *Sox9*^*iresCreERT2/+*^*; Sox2*^*fl/+*^*; p27*^*−/−*^ compared to *Sox9*^*iresCreERT2/+*^*; p27*^*−/−*^ (Fig. 5 *B*, *Lower*), and there were fewer SCs upon *Sox2* reduction (*SI Appendix*, Fig. S5*B*). SOX2 staining intensity in SCs was also reduced (Fig. 5*F* and *SI Appendix*, Fig. S5*C*). Furthermore, we observed a significant reduction of proliferation in both SCs and melanotrophs in 6-mo-old *Sox9*^*iresCreERT2/+*^*; Sox2*^*fl/+*^*; p27*^*−/−*^ IL compared to *Sox9*^*iresCreERT2/+*^*; p27*^*−/−*^ samples (Fig. 5 *C*, *D*, and *F*). In addition, formation of ectopic vascular structures is significantly decreased (Fig. 5 *E* and *F*).

Altogether these results indicate that, while SCs are not themselves giving rise to melanotroph tumors in *p27*^*−/−*^ IL, SOX2 activity in the SCs and consequently the SCs themselves are required for tumorigenesis in *p27*^*−/−*^ IL.

### Characterization of the Transcriptional Consequences of Altering *Sox2* Levels in *p27*^*−/−*^ IL SCs.

**Fig. 6.**
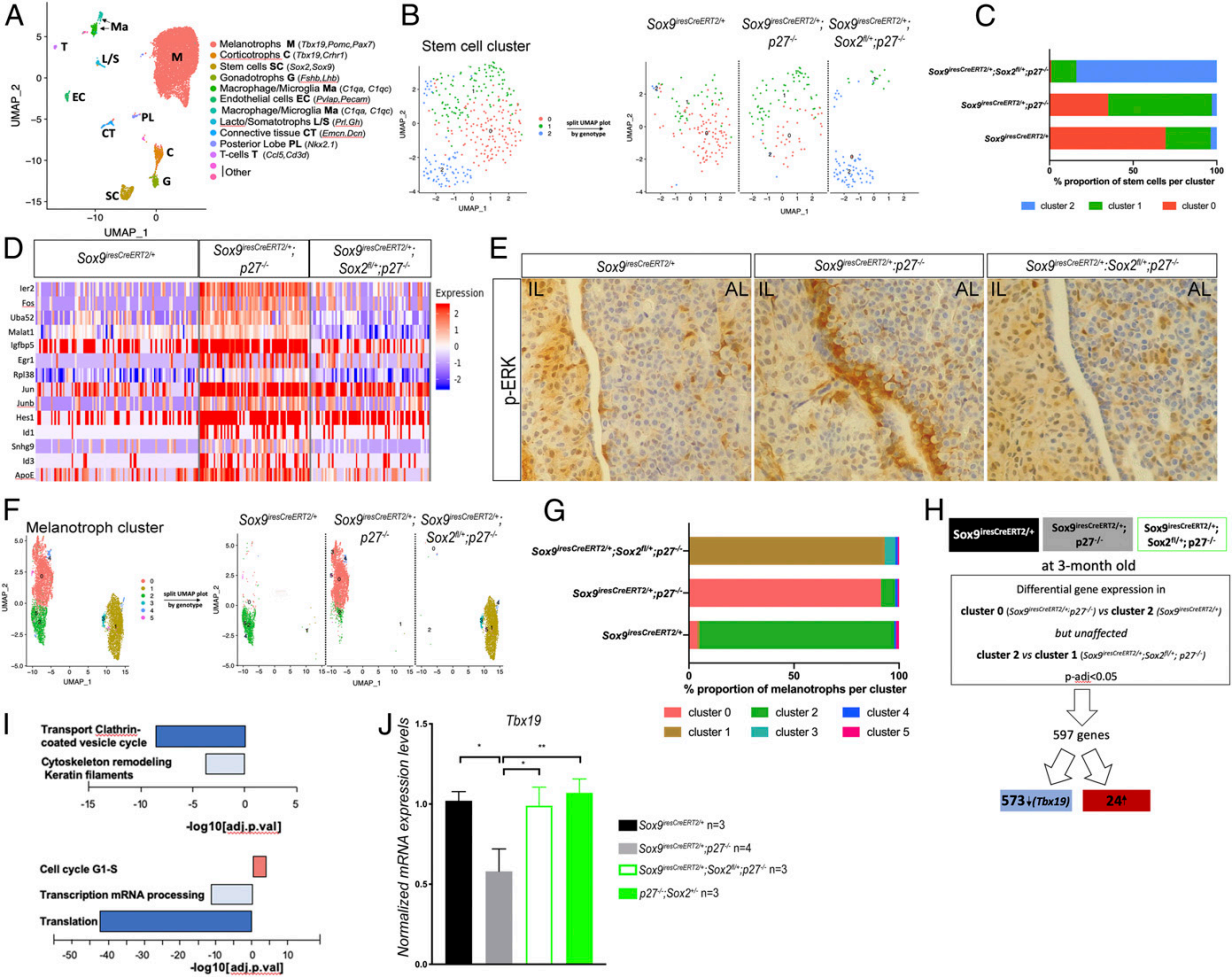
Characterization of the transcriptomic effects of *Sox2* dosage reduction in SCs in *p27*^*−/−*^ IL by single cell analysis. (*A*) Identification of clusters in integrated UMAP. (*B*) Subclustering of the stem cell fraction (0.9 resolution) shows that segregation only partially correlates with genotype (*Right*). (*C*) Representation of the proportion of cells of the indicated genotype in each cluster, *Sox9*^*iresCreERT2/+*^ (*n* = 137 cells), *Sox9*^*iresCreERT2/+*^*; p27*^*−/−*^ (*n* = 104 cells), and *Sox9*^*iresCreERT2/+*^*; Sox2*^*fl/+*^*; p27*^*−/−*^ (*n* = 82 cells). (*D*) Heatmap showing expression of 14 genes selected out of 36 differentially expressed genes (adjusted *P* value <0.05) in *Sox9*^*iresCreERT2/+*^*; p27*^*−/−*^ compared with *Sox9*^*iresCreERT2/+*^ or *Sox9*^*iresCreERT2/+*^*; Sox2*^*fl/+*^*; p27*^*−/−*^. (*E*) Immunohistochemistry for p-ERK on *Sox9*^*iresCreERT2/+*^, *Sox9*^*iresCreERT2/+*^*; p27*^*−/−*^, and *Sox9*^*iresCreERT2/+*^*; Sox2*^*fl/+*^*; p27*^*−/−*^ pituitaries. While the MAPK/ERK pathway seems overactive in *p27*^*−/−*^ SCs, p-ERK levels return to normal levels upon deletion of one copy of *Sox2* in SCs. (*F*) Subclustering of the melanotroph fraction (0.2 resolution) shows that segregation correlates with genotype (*Right*). (*G*) Representation of the proportion of cells of the indicated genotype in the most abundant cluster, *Sox9*^*iresCreERT2/+*^ (*n* = 1,272 cells), *Sox9*^*iresCreERT2/+*^*; p27*^*−/−*^ (*n* = 3,561 cells), and *Sox9*^*iresCreERT2/+*^*; Sox2*^*fl/+*^*; p27*^*−/−*^ (*n* = 3,006 cells). (*H*) Strategy followed for scRNAseq analysis of *Sox9*^*iresCreERT2*/+^; *p27*^−/−^ vs. *Sox9*^*iresCreERT2*/+^ or/and *Sox9*^*iresCreERT2*/+^; *Sox2*^*fl*/+^; *p27*^−/−^ melanotrophs. (*I*, *Upper*) Pathway analysis on genes differentially expressed (adjusted *P* value <0.05) in melanotrophs following the strategy delineated in *I*. Pathways associated with secretory function and cytoskeleton are down-regulated in *Sox9*^*iresCreERT2/+*^*; p27*^*−/−*^ cells. (*Bottom*) Process network analysis performed on genes differentially expressed between *Sox9*^*iresCreERT2/+*^*; p27*^*−/−*^ and *Sox9*^*iresCreERT2/+*^*; Sox2*^*fl/+*^*; p27*^*−/−*^ melanotrophs (adjusted *P* value <0.05). Processes associated with translation and transcription are down-regulated in *Sox9*^*iresCreERT2/+*^*; p27*^*−/−*^ melanotrophs, while genes promoting cell cycle progression are up-regulated. (*J*) RT-qPCR analysis of Tbx19 levels in 5- to 12-mo-old *Sox9*^*iresCreERT2*/+^, *Sox9*^*iresCreERT2*/+^; *p27*^−/−^, *Sox9*^*iresCreERT2*/+^; *Sox2*^*fl*/+^; *p27*^−/−^, and *p27*^−/−^; *Sox2*^+/−^ IL. Tbx19 levels are increased in *Sox9*^*iresCreERT2*/+^; *Sox2*^*fl*/+^; *p27*^−/−^ (0.99 ± 0.20, *n* = 3, *P = 0.02) and *p27*^−/−^; *Sox2*^+/−^ (1.07 ± 0.15, *n* = 3, **P = 0.0044) vs. *Sox9*^*iresCreERT2*/+^; *p27*^−/−^ (0.58 ± 0.28, *n* = 4).

To analyze the role of SOX2 in SCs, we performed single cell RNA sequencing analysis (scRNAseq) on ILs from 3-mo-old *Sox9*^*iresCreERT2/+*^, *Sox9*^*iresCreERT2/+*^*; p27*^*−/−*^, and *Sox9*^*iresCreERT2/+*^*; Sox2*^*fl/+*^*; p27*^*−/−*^ animals, where Cre activity had been induced at birth. Dataset clustering was performed by generating uniform manifold approximation and projection (UMAP) plots (Fig. 6*A* and *SI Appendix*, Fig. S6*A*). The three datasets were integrated and clusters identified according to the expression of known DEGs, and by comparison with published studies (24, 31). The major cell cluster corresponds to melanotrophs as expected (*Tbx19*, *Pomc*, and *Pax7* positive). The SC cluster (*Sox2* and *Sox9* positive) is proportionally larger in our IL dataset in comparison with studies performed on the whole pituitary (24, 31). We independently reanalyzed the SC (Fig. 6 *B* and *C*) and melanotroph clusters (Fig. 6 *F* and *G*). Within each cell type we observed that cells were mostly, but not perfectly, clustered according to their genotype. We thus aimed at examining DEGs between the clusters to characterize the direct effect of *Sox2* dosage on SCs, and the consequences on melanotrophs. More precisely, we analyzed DEGs in *Sox9*^*iresCreERT2/+*^*; p27*^*−/−*^ compared to *Sox9*^*iresCreERT2/+*^ and *Sox9*^*iresCreERT2/+*^*; Sox2*^*fl/+*^*; p27*^*−/−*^.

In SCs, (Fig. 6 *B* and *C*) 36 DEGs were identified by comparing subcluster 1 (which has the highest proportion of *p27*^*−/−*^ cells) to the two other subclusters (Dataset S3). Thirty of the DEGs were up-regulated in subcluster 1, and among them, 14 have been associated with tumorigenesis (Fig. 6*D*). Among these, 5 are immediate early response genes (*Ier2*, *Fos*, *Junb*, *Jun*, and *Egr1*) that can be activated by the MAPK pathway. This pathway was also activated in our *p27*^*−/−*^ bulk RNAseq dataset (Fig. 1*D*). In embryonic pituitary progenitors, activation of the MAPK pathway is associated with expansion of this compartment (32). We thus performed immunohistochemistry for phosphorylated ERK and observed a clear and specific up-regulation of the signal in *p27*^*−/−*^ SCs, in contrast with both control and *Sox9*^*iresCreERT2/+*^*; Sox2*^*fl/+*^*; p27*^*−/−*^ samples (Fig. 6*E*). This suggests that SOX2 positively regulates activity of the pathway in SCs, either directly or indirectly, and this correlates with tumorigenesis.

Subclustering of the melanotrophs, which were more numerous than the SCs, was better associated with the different genotypes (Fig. 6 *F* and *G*). In agreement with the absence of tumorigenesis, there was a reduction in melanotroph numbers in *Sox9*^*iresCreERT2/+*^*; Sox2*^*fl/+*^*; p27*^*−/−*^ compared to *Sox9*^*iresCreERT2/+*^*; p27*^*−/−*^, but these were still more numerous than in wild type, in agreement with the hyperplasia observed in compound mutants (Fig. 6*F*). Furthermore, while melanotrophs are genetically equivalent in *Sox9*^*iresCreERT2/+*^*; p27*^*−/−*^ and *Sox9*^*iresCreERT2/+*^*; Sox2*^*fl/+*^*; p27*^*−/−*^ samples, their transcriptome is clearly different, further confirming the SOX2-dependent effect of SCs on melanotrophs. There were 597 DEGS in cluster 0 (*Sox9*^*iresCreERT2/+*^*; p27*^*−/−*^) melanotrophs compared to clusters 2 (*Sox9*^*iresCreERT2/+*^) and 1 (*Sox9*^*iresCreERT2/+*^*; Sox2*^*fl/+*^*; p27*^*−/−*^) (Fig. 6*H*). Pathway analysis of the 573 down-regulated genes in *Sox9*^*iresCreERT2/+*^*; p27*^*−/−*^ melanotrophs revealed that vesicular and endosomal traffic, and cytoskeleton remodeling, as we observed in our bulk analysis (Figs. 1 *C* and *D* and 2*B*), were predominantly affected, suggesting that the differentiated, secretory phenotype of melanotrophs was restored upon reduction of SOX2 in SCs (Fig. 6 *I*, *Upper*). In agreement with this hypothesis, *Tbx19* was among the down-regulated genes in *Sox9*^*iresCreERT2/+*^*; p27*^*−/−*^ melanotrophs. Recovery of normal expression levels of *Tbx19* upon *Sox2* down-regulation in SCs was further validated by quantitative PCR (Fig. 6*J*). Finally, pairwise comparison between clusters 0 and 1, comprising *Sox9*^*iresCreERT2/+*^*; p27*^*−/−*^ and *Sox9*^*iresCreERT2/+*^*; Sox2*^*fl/+*^*; p27*^*−/−*^ cells, respectively, revealed a significant enrichment in process networks associated with translation and transcription (Fig. 6 *I*, *Bottom*), suggesting reduced levels of both transcription and translation in *p27*^*−/−*^ cells, restored in melanotrophs upon reduction of *Sox2* levels in SCs. SCs and CSCs are known to have reduced levels of translation. Moreover, SOX2 was recently shown to be directly involved in alterations in the rate of protein synthesis in a mouse model of squamous tumor initiating cells (33). This suggests that SOX2 may play a similar role in *p27*^*−/−*^ melanotrophs, acting as a repressor of translation and transcription of genes related to differentiation.

Altogether these data show that SOX2 up-regulation in *p27*^*−/−*^ SCs results in the induction of a dedifferentiated, protumoral phenotype in melanotrophs. In SCs, SOX2 overexpression leads to increased MAPK pathway activation, which correlates with and may mediate the role of SOX2 in tumorigenesis, at least in *p27*^*−/−*^ IL.

## Discussion

**Fig. 7.**
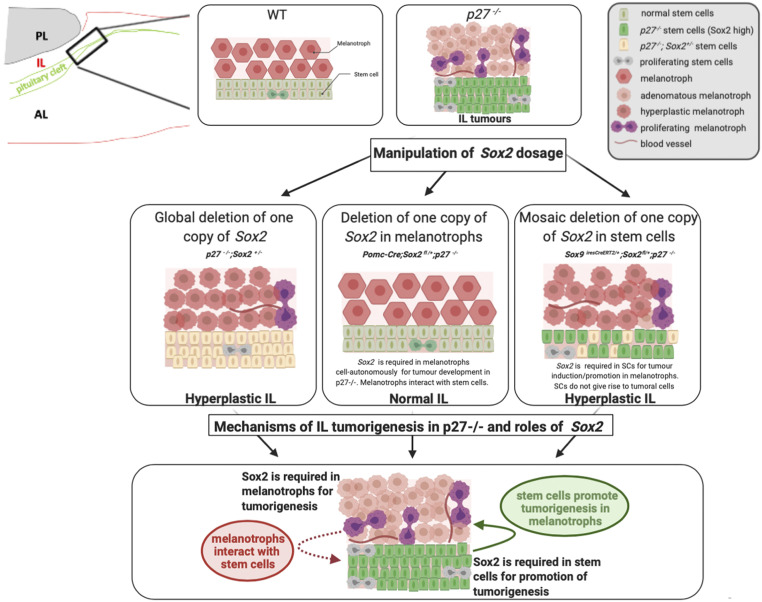
Model recapitulating the consequences of *Sox2* dosage modulation during IL *p27*^*−/−*^ tumorigenesis and its proposed roles. In normal IL, SOX2 is expressed at high levels in SCs and at low levels in melanotrophs. In *p27*^*−/−*^ IL, melanotroph tumors develop with increased SC proliferation. SOX2 levels are increased in both cell types, while expression of TBX19 and POMC is reduced in melanotrophs. Removal of one copy of *Sox2* in *p27*^*−/−*^ animals *(p27*^*−/−*^*; Sox2*^*+/−*^*)* results in impairment of tumorigenesis, while hyperplasia is still observed. Conditional deletion of *Sox2* in melanotrophs (*Pomc-Cre; Sox2*^*fl/+*^*; p27*^*−/−*^) prevents hyperplasia and tumor formation; therefore, SOX2 is required cell autonomously for tumor formation (Fig. 4). Furthermore, modulation of SOX2 levels in SCs in this model suggests that melanotrophs interact with SCs (dotted arrow). Conditional deletion of *Sox2* in SCs (*Sox9*^*iresCreERT2*^; *Sox2*^*fl/+*^*; p27*^*−/−*^) also impairs tumorigenesis, reduction of *Tbx19* levels, and SC overproliferation but hyperplasia is still observed (Figs. 5 and 6). Therefore SCs promote tumor formation and loss of differentiated features, while not giving rise to tumorigenic cells themselves. This tumor-promoting role of SCs (arrow) depends on SOX2. Therefore SOX2 is required both in melanotrophs and SCs for respectively cell-autonomous and tumor-promoting functions following loss of P27. This model was created with BioRender.com.

The regulation of *Sox2* expression is extremely complex (34) and variation of its expression levels has contrasting consequences in cancers (16). Here we have further dissected the role of SOX2, and also demonstrated the importance of *Srr2* for the SOX2-P27 interaction. We have shown that SOX2 is required independently both in melanotrophs and SCs for IL tumors to develop in *p27*^*−/−*^ animals (see model, Fig. 7). The pleiotropic, protumorigenic effects of SOX2 in distinct cell types in the absence of P27 demonstrate that it is a crucial mediator of tumorigenesis and hence, a good target for antitumoral treatments. However, we also show that SOX2 only appears to be a relevant target in a subset of tissues when P27 is lost or its expression decreased, such as the pituitary and duodenum.

Understanding the reasons behind the tissue specificity for the SOX2-P27 interaction could help to define preexisting factors favoring tumorigenesis. Our results suggest that derepression of *Sox2* in the absence of P27 leads to IL tumors. However, this happens only in some cell types, while expression of *p27* is ubiquitous, likely illustrating the multiple antiproliferative roles of P27 that are distinct from *Sox2* repression (35, 36). Differential expression of P27 interactors and/or targets, and of other cell cycle repressors, may explain tissue-specific features of the *p27*^*−/−*^ phenotype.

In pituitary endocrine cells, SOX2 expression appears to be a prerequisite for tumoral development in absence of *p27*, since melanotrophs are the only such cell type where SOX2 is detectable (19). While melanotrophs are also uniquely characterized by their nonproliferative status (6), levels of P27 are unexpectedly relatively low in melanotrophs. This suggests that in *p27*^*−/−*^ mutants, an active *Sox2* locus normally under P27 control, coupled with a relaxed control of the cell cycle renders melanotrophs particularly prone to tumorigenesis. In agreement, independent deletion of genes encoding for different negative cell cycle regulators show that the IL is particularly sensitive to tumoral development (7). Furthermore derepression of *Sox2* is also observed in *pRb*^*+/−*^ pituitary tumors (37). It would be interesting to examine whether SOX2 is required for IL tumorigenesis in other cell cycle regulator mutants (7). Conversely we can now screen for cell types satisfying the relevant criteria, including low cell turnover, low negative cell cycle regulator levels, and presence of SOX2, and examine for tumorigenic potential.

To further characterize the molecular mechanisms involved in the SOX2-P27 interaction, we have deleted *Srr2*. SOX2 itself, c-myc, and members of the POU transcription factor family bind to *Srr2* to promote expression of the gene (38). Beside P27 (15), other negative regulators of *Srr2* have been characterized such as P21 in neural SCs (39) and pRb in the pituitary (37). Analysis of *p27*^*−/−*^*; Srr2*^*del/del*^ animals shows that the repressive action of P27 is mediated by *Srr2* and in absence of *p27*, Srr2 activators drive *Sox2* expression; this then leads to pituitary tumors and retina defects (see model, *SI Appendix*, Fig. S3*E*). Notably, loss of *Srr2* affects cell proliferation exclusively in *p27*^*−/−*^ SCs, suggesting that the role of the enhancer is more important in SCs, in agreement with a role for these in promoting the melanotroph tumors.

Our study highlights common and cell-specific roles of SOX2 during tumorigenesis. Both in *p27*^*−/−*^ melanotrophs and SCs, SOX2 up-regulation is associated with overproliferation. The association of SOX2 with dedifferentiation and overproliferation has been observed in many tumors (16), as we also observe here in melanotrophs. In pituitary SCs, our results suggest that this overproliferation is linked to MAPK overactivation, something that has been shown in embryonic pituitaries expressing an activated form of *Braf* (32). How SOX2 derepression induces MAPK activation is less clear; MAPK pathway activation has been linked to induction of *Sox2* expression (40), but the molecular mechanisms undelying the converse have not been characterized. It would be of interest to determine whether this SOX2-MAPK interaction is relevant in other contexts. While SOX2 has been associated with CSC properties in some other tumors (41), we report here its requirement in SCs for these to promote tumorigenesis in neighboring melanotrophs. Manipulation of MAPK in SCs in *p27*^*−/−*^ animals is now required to characterize their effect on SCs and melanotroph transformation.

In conclusion, our study reveals how *Sox2* derepression, independently in two adjacent cell types, underlies tumorigenesis in one of them. Moreover, our work reveals the existence of reciprocal interactions between melanotrophs and flanking SCs to orchestrate tumor development. This highlights the complexity of the mechanisms triggering tumorigenesis and the central role of SOX2 in this process. Furthermore pituitary SCs, which are not tumoral, but have a tumor-inducing activity, represent good antitumoral treatment targets, highlighting the importance of detailed characterization of mechanisms underlying tumorigenesis to decipher possible antitumor strategies.

## Materials and Methods

All experiments carried out on mice were approved under the UK Animal (scientific procedures) Act (project licenses 80/2405 and 70/8560). Detailed protocols and mouse strains can be found in *SI Appendix*, *Supplementary Material and Methods*.

## Supplementary Material

Supplementary File

Supplementary File

Supplementary File

Supplementary File

## Data Availability

RNA sequencing datasets have been deposited in Gene Expression Omnibus (GSE152010). All study data are included in the article and/or supporting information.
