# Supplementary material for: SOX2 is required independently in both stem and differentiated cells for pituitary tumorigenesis in p27-null mice
Source: Proc Natl Acad Sci U S A. 2021 Feb 11;118(7):e2017115118. doi: 10.1073/pnas.2017115118 (PMC7896314; doi:10.1073/pnas.2017115118)
Supplement: Supplementary File [file pnas.2017115118.sapp.pdf]

Supplementary Material and Methods

Mice

Cdkn1b^{tm1Mlf} (*p27*$^{-}$) (8), *Sox9*$^{tm1(cre/ERT2)Haak}$ (*Sox9*^{iresCreERT2}) (29), *Tg(PomC-Cre)Dro* (*Pomc-Cre)* (5), *Sox2*^{tm1Lpev} (Sox2^{GFP}), *Sox2*^{tm2Lpev} (Sox2^{fl}) (42) and *Gt(ROSA)26Sor*$^{tm1(EYFP)Cos}$ (*Rosa26*^{ReYFP}) (43) were maintained on mixed background. Experiments were either performed using littermates or animals with comparable backgrounds.

Cre activity was induced by tamoxifen administration for 3 consecutive days at 5mg/25g body weight /day. Pituitaries were harvested after tamoxifen treatment, as indicated in each experiment. Genotyping was performed by Transnetyx®.

Detailed information on animals used/experiment is available in Supplementary Table 4.

Generation of Pomc-CreERT2 transgenic mice and and *Sox2* $^{tm1(Guide1/1:SRR2)RLB}$ mice

Tg(Pomc-cre/ERT2)Rlb (*Pomc-CreERT2*) mice were generated by inserting a 543bp (-480/+63) region of the *Pomc* promoter (5) upstream of the sequence coding for iCreERT2. *Pomc-CreERT2* animals were obtained by standard pronuclear injection. Tissue-specific activity of Pomc-CreERT2 was confirmed by crossing transgene positive animals with *Rosa26*$^{ReYFP/ReYFP}$ mice; one founder was chosen to establish the strain.

The *Sox2* $^{tm1(Guide1/1:SRR2)RLB}$ allele was generated using the CRISPR/Cas9 technology. Single guide RNAs (sgRNAs) were designed (www.crispr.mit.edu) to induce deletion of the mouse SRR2 enhancer (81 bp), situated 4kb downstream of *Sox2* (UCSC mm9 genome chr13: 34,552,926 - 34,553,007). Two pairs of guides were designed: pair 1(G1_5'+G1_3') and pair 2 (G4_5'+G5_3') (Supplementary Table 1) and independently injected into zygotes with Cas9 mRNA as previously described (44) to produce two different mouse strains with the same *Srr2* deletion, in order to discriminate between the effects of the desired mutation, which would be common to both guides, and potential off-target effects of the guides which would be in contrast specific to a particular pair. Founders were genotyped for SRR2 deletion by PCR and mosaicism estimated using the MiSeq system (Illumina) (Supplementary Table 2, adaptor sequence in bold). Two founders were chosen to establish two independent strains.

Immunofluorescence, EdU staining, image acquisition and pre-processing

Immunofluorescence was performed as previously described (45). Mice were perfused with 4% w/v paraformaldehyde in phosphate-buffered saline (PBS), pituitaries harvested and cryosectioned at 12 μm. Sections were blocked with blocking solution (10% v/v donkey serum in PBS/0.1% v/v Triton X-100; PBST) for 1 hr, then incubated with primary antibodies in 10% blocking solution overnight at 4°C. Primary antibodies were used at the following dilutions: rat anti-GFP (Nacalai Tesque) at 1:1000; rabbit anti-POMC (NHPP) at 1:500; mouse anti-POMC (46) 1:500, Mouse anti-PAX7 (DSHB) 1:100, goat anti-SOX2 (Immune System) at 1:300, rabbit anti-SOX9 (gift from F. Poulat, Institut de Génétique

Humaine, Montpellier) at 1:300, rat anti-CD31 (47) 1:50, p27kip C19 (SantaCruz) 1:300, rabbit anti-ki67 (Abcam) 1:1000. Sections were washed in PBST then incubated for 1 hr at room temperature with the corresponding anti-rat, anti-goat or anti-rabbit secondary antibody conjugated to Alexa-Fluor 488, 555, 594 or 647 in 10% blocking solution with 1 μM 4',6-diamino-2-phenylindole (DAPI).

Cell proliferation was analysed following a one-hour EdU pulse in E18.5 embryos (30µg/g body weight), or two intraperitoneal daily injections for 3 consecutive days in adult. Adult pituitaries were harvested at day 4. EdU incorporation was detected using the Click-iT EdU imaging kit following the manufacturer instructions (Thermo Fisher Scientific). Sections were washed with PBST and mounted using Aqua-Poly/Mount (Polysciences, Inc., Warrington, PA, USA).

Images were acquired using a Leica SPE confocal microscope or Olympus VS120 Slide Scanner. Settings were established during the initial acquisition. All images taken from the Leica SPE confocal microscope were pre-processed using ImageJ (maximum z-projection) and the ones taken from VS120 Slide Scanner were processed using Qu-path.

Immunohistochemistry

Pituitary glands were fixed in 10% buffered formaldehyde for 16 hours and embedded in paraffin. For staining, 4µm sections were de-paraffinized using xylene and rehydrated through a graded series of ethanol. Antigen retrieval was performed for 20 min at high temperature in either 0.01M citrate buffer (pH6) or Tris-EDTA (10mM Tris base, 1mM EDTA solution, pH9), depending on the antibody. The following antibodies were used: Sox2 (AF2018, R&D), 1:100, on sections treated with antigen retrieval buffer (Ventana) for 48 minutes, followed by a one hour primary antibody incubation. IHC was performed on the Discovery Ultra Ventana platform (Roche). P-ERK1/2 (4370, Cell Signalling Technology) 1:100 O/N at 4ºC, antigen retrieval was performed in the microwave for 23 minutes with 0.01M citrate buffer pH6. Goat anti-rabbit secondary antibody (BA-1000, Vector) was incubated 1:250 for 45 minutes at RT and then signal amplification and HRP detection were performed using the ABC kit (Vectorlabs) for 30 minutes at RT. This IHC was performed manually. Samples were blocked using 1% BSA and incubated overnight at 4°C with the desired antibody, or in blocking buffer for controls. Finally, slides were incubated with the secondary antibody for one hour and washed three times with PBS. For colorimetric staining with diaminobenzidine (DAB) slides were incubated with peroxidase substrate and mounted.

Cell Countings

For proliferation assays quantification, three different fields were chosen on different sections (sections always include IL, SCs flanking the cleft and AL). Within these, the numbers of SOX2-positive, SOX9-positive, EdU-positive, and SOX2;EdU;SOX9 triple-positive cells, representing proliferative stem cells, or SOX9 negative, SOX2;EdU double-

positive cells, representing proliferative melanotrophs, were counted blindly and manually.
In EdU injected *Pomc-CreERT2; Sox2*$^{fl/+}$*;p27*$^{-/-}$*;Rosa26*$^{ReYFP/+}$ mutants, a total of around 1000 PAX7 positive cells was counted from fields randomly chosen and encompassing the whole IL. The number of EdU;PAX7-double-positive or EdU;PAX7;eYFP-triple-positive cells was counted blindly and manually, and the IL surface area was determined using the Fiji software.
The Fiji software was used to pseudocolor the different channels in unprocessed micrographs.
The Qupath software (48) was also used for image analysis. Briefly, areas of interest were defined after POMC immunostaining for the intermediate lobe, or SOX2;SOX9 for the SC layer. Within the areas of interest, the centre of cell nuclei was then identified as maxima in a filtered DAPI image. Nuclear boundaries were assigned by a propagation algorithm, and then expanded by ~1 micron to define sampling areas. The following data were then recorded: (i) average pixel intensities for each data channel over each sampling area, representing one cell, (ii) the size of the sampling areas; and (iii) the number of positive cells for each data channel over each sampling area.

Pituitary Dissociation for FACS, sc-RNAseq and Sphere Assay

Pituitaries were harvested. The anterior lobe was separated from the intermediate and posterior lobes by cutting through the cleft. The posterior lobe was then removed and the anterior and intermediate lobes incubated separately in a solution of papain (10108014001, Sigma-Aldrich, 1mg/ml in HBSS) for 15 min at 37ºC in presence of 10µg/ml of both DNAse (10104159001, Thermo Scientific) and Rock Inhibitor (M1817, Abmole Bioscience). The papain solution was then removed, and mechanical dissociation was performed on ice in pituisphere medium (18). Pituispheres were derived as previously described (18) and spheres counted manually and blindly after 1 week in culture.

mRNA extraction and reverse transcriptase-PCR (rt-qPCR)

Total mRNA was extracted from dissected IL using the RNeasy Micro kit (Qiagen) according to the manufacturer's protocol. The extracted RNA was reverse-transcribed into cDNA using the Superscript VILO cDNA synthesis kit (Thermo Fisher Scientific, Figure 2) or SMART-Seq v4 Ultra Low Input RNA Kit (Takara Bio USA, Figure 5) according to the manufacturer's protocol.

Quantitative real-time PCR (RT-qPCR)

Each sample was assayed in technical duplicate with each tube containing diluted template cDNA, 250 nM primers and 1xAbsoluteSybrGreen ROX mix (Thermo Fisher Scientific). Each sample was assayed for the genes of interest together with the reference housekeeping gene *Gapdh* (Supplementary Table 3). Relative expression of the genes of interest was

calculated by normalisation of the detected expression value to the geometric mean of the reference genes using the ΔΔCt method (49). Data is shown as mean±SEM with the number of biological samples indicated in each figure. Sidak's (Figure 1I) and Tukey's multiple comparison tests (Figure 2H and 5K) were used to assess significance of the data.

Radioimmunoassay (RIA)

Pituitaries were homogenized in phosphate-buffered saline and hormonal contents measured by RIA (50) using National Hormone and Pituitary Program reagents kindly provided by A.L. Parlow for ACTH and GH and using alpha MSH RIA kit (RB303-Invitech).

In-situ hybridization (RNAscope)

Fluorescent in situ hybridisations were performed manually using RNAscope (Multiplex Fluorescent Reagent Kit v2, 320293) on cryosections according to the manufacturer's protocol. Images were analysed and quantified using the Fiji package.

Statistical analyses

Statistical analyses were performed using Prism v.8.0c (GraphPad Software, USA). To examine significance of the data, tests were selected according to the experiment analysed. For survival curves generated using the Kaplan Meier method, log-rank tests were applied (Fig.2B). When comparing two groups of values with normal distribution, unpaired t-test was performed (Figure; 1H, 3H, 3I and in Supplementary figure;1B & 4B). Angular transformation was applied to compare percentages followed by unpaired t-test (Figure; 1L,1M, 2E and 3D. When comparing two groups of values where the distribution was non-parametric, Mann Whitney test was performed (Figure; 1C (upper graph), 1G, 1J). For multiple comparisons, analysis of variance (ANOVA) was implemented; Sidak's test was performed when comparing two groups (genotype) and differences between compartments (AL, SC cleft or IL) in Figure; 1C (bottom panel),1K,1O. To analyse larger groups, Tukey's multiple comparison test was implemented (Figure 2G, 2M & O, 3C,3K,4C,4D,4E and Supplementary Figure;2B,4A). All results are represented as means ± standard deviation (SD) for raw data and means ± standard error of the mean (SEM) for graphs. Standard significance levels were used: *$p<0.05$, **$p<0.01$, ***$p<0.001$, ****$p<0.0001$.

RNA sequencing sample preparation

Bulk RNA sequencing. RNA was extracted from dissected male and female IL or FAC sorted cells using the RNeasy Micro kit (Qiagen) according to the manufacturer's protocol. RNA quality was assessed using the Agilent RNA 6000 Pico Kit (Agilent Technologies). cDNA was generated using Ovation RNA-seq System V2 (Tecan, 7102-A01), libraries were constructed using Ovation Ultralow System V2 (Tecan, 0344NB-A01) according to the manufacturer's instructions. Libraries were quantified using the TapeStation (Agilent)

and pooled in equimolar proportions. Library were sequenced on an Hiseq4000 (Illumina), to achieve an average of 25 million reads per sample.

Single-cell RNA sequencing. Male and female IL cells were dissociated as described above. Libraries were generated using Chromium Single Cell 3' kit v3 (10x genomics, 100092) according to the manufacturer's instructions. Both cDNA and libraries were quantified using the TapeStation (Agilent) and sequenced on an Hiseq4000 (Illumina), to achieve an average of 50,000 reads per cell.

Detailed aims and animal information is available in Supplementary Table 5.

Bioinformatics analysis

Bulk RNA sequencing. The sequencing was performed on biological duplicates or triplicates for each data point. The RSEM package (version 1.3.30) (51) was used in conjunction with the STAR alignment algorithm (version 2.5.2a) (52) for the mapping and subsequent gene-level counting of the sequenced reads with respect to Ensembl mouse GRCm.38.89 version transcriptome. All parameters for RSEM were run as default except "–forward-prob" which was set to 0.5. Normalisation of raw count data and differential expression analysis was performed with the DESeq2 package (version 1.18.1) (53) within the R programming environment (version 3.4.3) (54). Differentially expressed genes were defined as those showing statistically significant differences between pairwise groups if the adjusted P value was less than 0.05 (FDR < 0.05). Differentially expressed genes were taken forward and their pathway and process enrichments were analysed using Metacore (https://portal.genego.com). A hypergeometric test was used to determine statistical enriched pathways and processes and the associated P-value was corrected using the Benjamini–Hochberg method.

Single-cell RNA sequencing. 10x CellRanger (version.3.0.2) was used to generate single cell count data for each genotype (*Sox9*$^{iCreERT2/+}$*;Rosa26*$^{ReYFP/+}$, *Sox9*$^{iCreERT2/+}$*;p27*$^{-/-}$ *Rosa26*$^{ReYFP/+}$ and *Sox9*$^{iCreERT2/+}$*;Sox2*$^{fl/+;}$*p27*$^{-/-}$ *Rosa26*$^{ReYFP/+}$) using a transcriptome built from the Ensembl mouse GRCm38 release 89. All subsequent analyses were performed in R v.3.6.0 using the Seurat (v3) package (55). Primary filtering was performed on each dataset by removing from consideration: cells expressing fewer than 50 genes and cells for which mitochondrial genes made up greater than 10% of all expressed genes. Each dataset was normalised using the' LogNormalize' function, with a scale factor of 10,000. The top 2000 highly variable genes were found using the 'FindVariableGenes' function and the data centred and scaled using the 'ScaleData' function. PCA decomposition was performed and after consideration of the eigenvalue 'elbow-plots, the first 20 components were used to construct Uniform Manifold Approximation Projection (UMAP) plots. Clusters relating to Melanotrophs and Stem Cells were identified, using the expression of known markers [melanotroph markers : Pomc, Pax7 and Pcsk2; stem cell markers : Sox2 and Sox9], and these clusters were integrated across the three genotypes (*Sox9*$^{iCreERT2/+}$*;Rosa26*$^{ReYFP/+}$, *Sox9*$^{iCreERT2/+}$*;p27*$^{-/-}$*Rosa26*$^{ReYFP/+}$ and *Sox9*$^{iCreERT2/+}$*;Sox2*$^{fl/+;}$*p27*$^{-/-}$ *Rosa26*$^{ReYFP/+}$) using Seurat 3's standard integration workflow.

Differentially expressed genes between clusters across genotypes were determined using the 'FindMarkers" function.

Data availability

The RNA-sequencing datasets have been deposited in the Gene Expression Omnibus with the grouped accession number GSE152010. The bulk RNAseq datasets are GSE152007 (*p27*$^{-/-}$ IL compared to wild-type, two- and seven-month old) and GSE152008 (*p27*$^{-/-}$ IL compared to *p27*$^{-/-}$ *;Sox2*$^{+/-}$ and wild-type, two- to three-month old); the single-cell RNA-sequencing datasets have been deposited with accession number GSE152009.

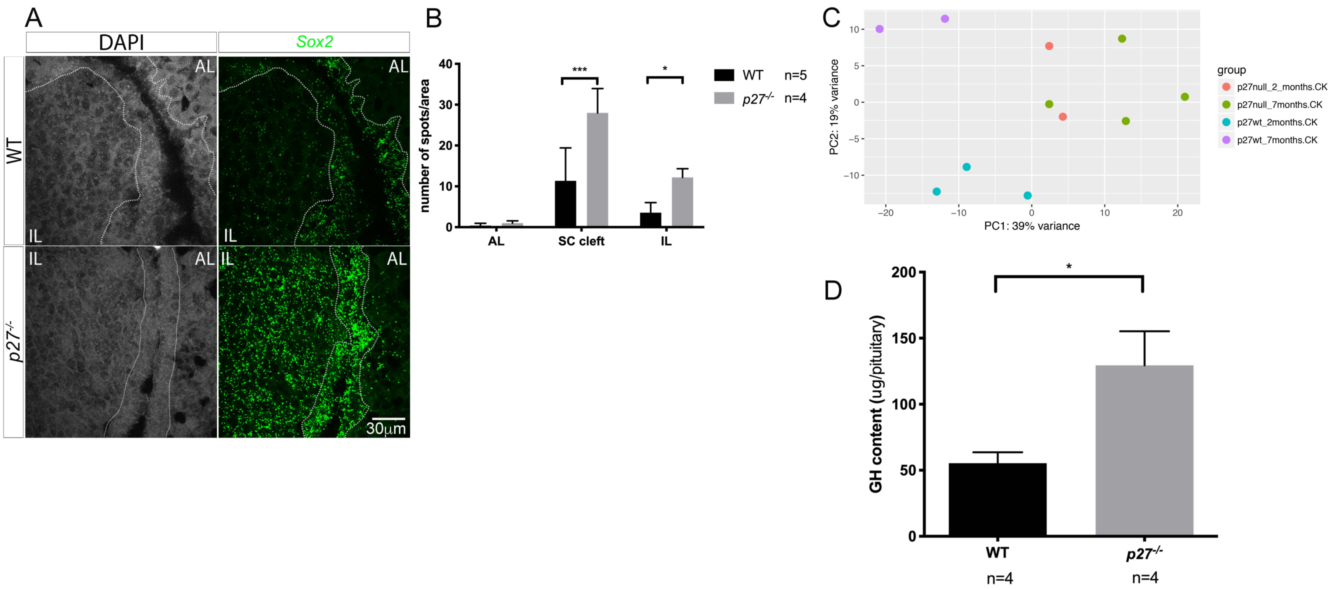

Fig. S1. Analysis of *p27*$^{-/-}$ pituitaries.

A) *In situ* hybridisation for *Sox2* in a 2-month old pituitary. In *p27*$^{-/-}$ IL higher levels of *Sox2* are observed in both melanotrophs and SCs .

B) Levels of SOX2 expression were quantified after *in situ* hybridisation, in the cell types indicated. Quantification after RNAscope show that in *p27*$^{-/-}$, SCs flanking the cleft express *Sox2* at higher levels (28±6) than and wt (11.4±8, ***p=0.0002). The same is observed in melanotrophs: *p27*$^{-/-}$ (12±2) and wt (3.5±2.5, *p=0.0134), (n=5-4 in each group).

C) Principal Component Analysis (PCA) plots of bulk RNA-seq data of 2- and 7-month old wildtype and *p27*$^{-/-}$ IL samples. There is a clear segregation of samples according to genotype, even before tumorigenesis (2 month-old). Furthermore while wild-type samples segregate according to age, *p27*$^{-/-}$ samples appear more similar, independently of the age of the animal.

D) GH content were measured by RIA in 2-month old male pituitaries. *p27*$^{-/-}$ pituitary glands contain higher GH levels (*p=0.0340, n=4 in each group) which is consistent with gigantism affecting these mutants.

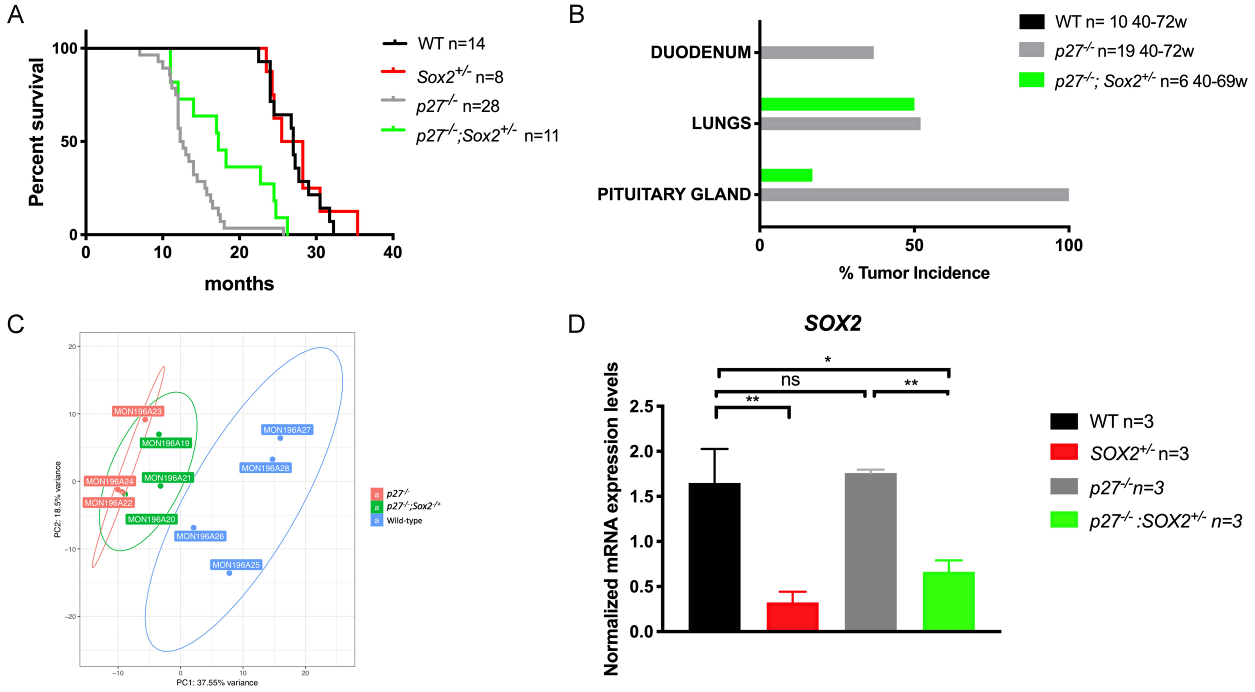

Fig. S2. Analysis of *p27$^{-/-}$;Sox2$^{+/-}$* and *p27$^{-/-}$;Srr2$^{del/del}$* pituitaries.

A) Kaplan-Meier survival curves for wt, *Sox2$^{+/-}$*, *p27$^{-/-}$* and *p27$^{-/-}$; Sox2$^{+/-}$* animals. *p27$^{-/-}$; Sox2$^{+/-}$* mutants survive longer (17.1 months, n=10) than *p27$^{-/-}$* mutants (12.5 months, n=28, *p<0.01), but still significantly less than wt (27 months, n=14,****p<0.0001).

B) Tumour incidence in the most affected organs in *p27$^{-/-}$* mice. *Sox2* heterozygosity results in a reduction of tumour incidence in the pituitary and duodenum but not in lungs.

C) PCA plots of bulk RNA-seq data of 2- to 3-month old wild-type, *p27$^{-/-}$* and *p27$^{-/-}$;Sox2$^{+/-}$* ILs. *p27$^{-/-}$;Sox2$^{+/-}$* samples are clustering between wild-type and *p27$^{-/-}$* ones.

D) RT-qPCR analysis of *Sox2* in wild-type, *Sox2$^{+/-}$* , *p27$^{-/-}$*,and *p27$^{-/-}$;Sox2$^{+/-}$* in 2- to 3-month old ILs. *Sox2* expression levels decrease in *Sox2$^{+/-}$* and *Sox2$^{+/-}$;p27$^{-/-}$* vs w*t or p27$^{-/-}$*. We did not observe an increase in *Sox2* levels in *p27$^{-/-}$* using this technique. This may be because IL comprises two cell types expressing *Sox2* at different levels: a high proportion of melanotrophs, expressing it at low levels, and SCs, expressing it at high levels. In *p27$^{-/-}$* animals there are many more melanotrophs than in wild-type, but these, despite *Sox2* derepression, are still expressing *Sox2* at lower levels than SCs (Fig.1A). The difference in proportion in both cell types between wildtype and mutant may explain the inability to observe *Sox2* derepression.

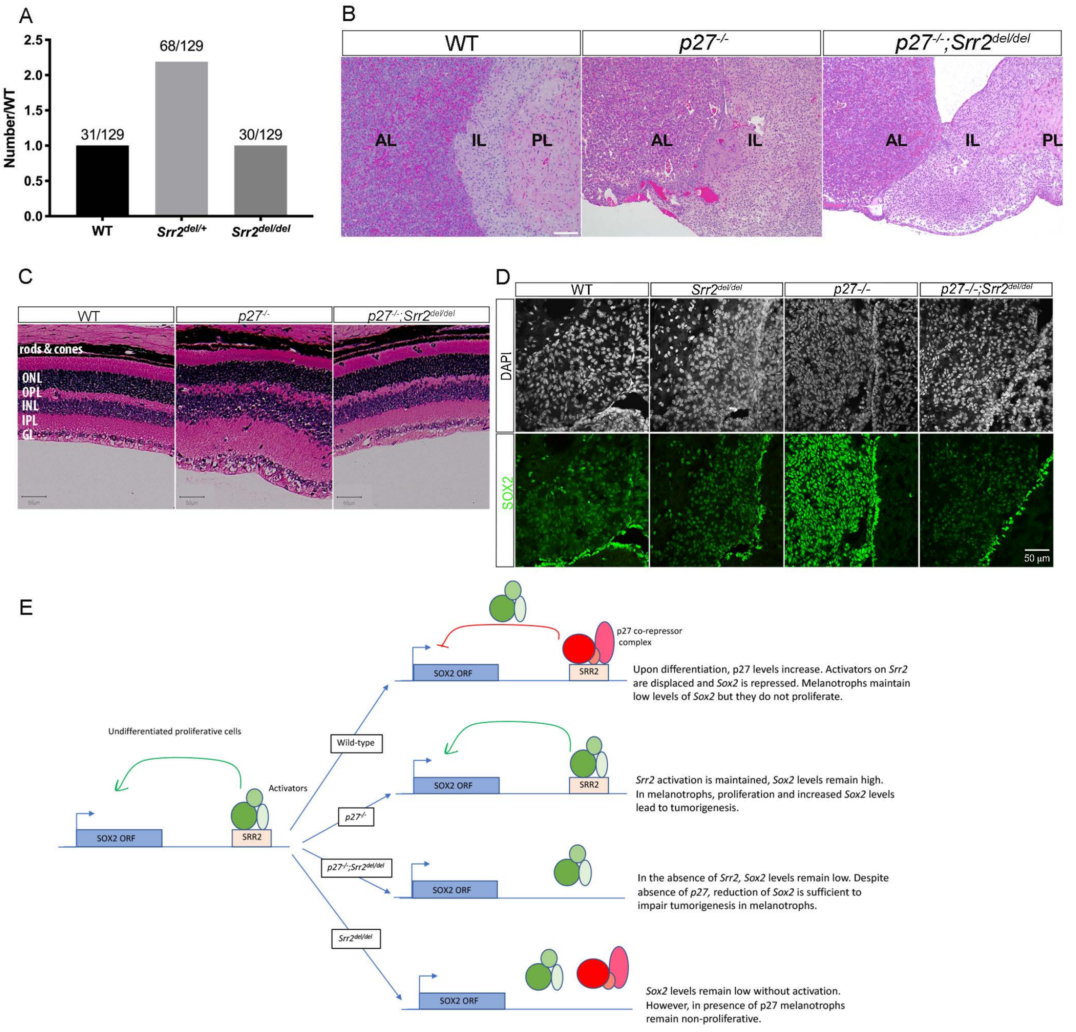

Fig. S3. Analysis of *p27$^{-/-}$;Srr2$^{del/del}$* pituitaries.

A) Genotypes of offspring obtained from *Srr2$^{del/+}$* heterozygous (XX) and (XY) intercrosses.

B) Histological H&E stained sections of 28 to 33-week old wt, *p27$^{-/-}$* and *p27$^{-/-}$;srr2$^{del/del}$* pituitaries. *p27$^{-/-}$;srr2$^{del/del}$* pituitaries show an hyperplastic but still reduced IL compared to *p27$^{-/-}$*. (Scale bar 100 microns)

C) Histological sections of H&E stained retinas. In *p27$^{-/-}$;srr2$^{del/del}$* retina, size and organization of the layers is improved compared to *p27$^{-/-}$*. ONL = outer nuclear layer; OPL= outer plexiform layer, INL = inner nuclear layer, IPL=inner plexiform layer, GCL= ganglion cell layer.

D) Immunofluorescence for SOX2 in 4 to 7-month old wt, *Srr2$^{del/del}$*, *p27$^{-/-}$* and *p27$^{-/-}$;Srr2$^{del/del}$* pituitary sections. SOX2 expression appears decreased *in p27$^{-/-}$;Srr2$^{del/del}$* IL.

E) Model illustrating the effects of p27 on Srr2 enhancer and consequences of their loss in melanotrophs. Srr2del/del animals do not phenocopy aspects of the p27-/- phenotype, which might have been expected if all the enhancer does is mediate repression of Sox2 by P27. However, in agreement with the phenotype of p27-/-; Srr2del/del animals, deletion of Srr2 prevents both repression and derepression of SOX2.

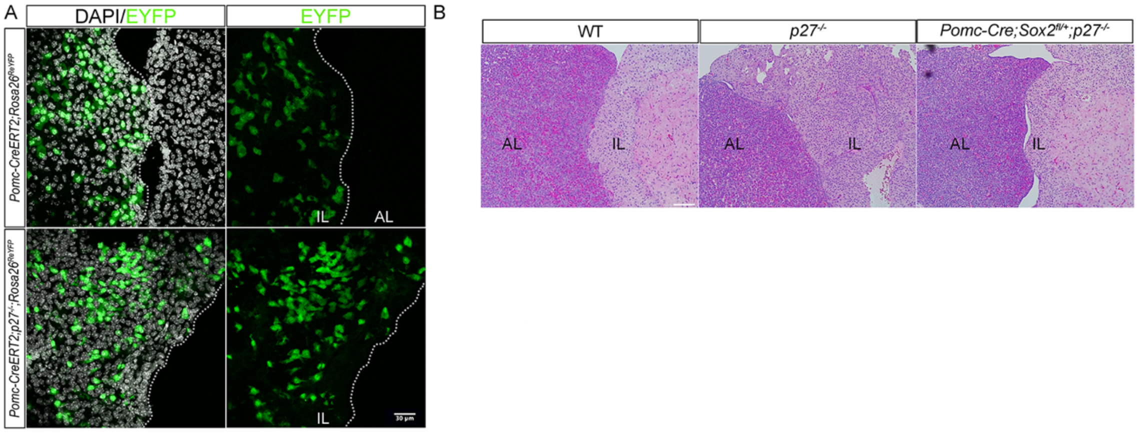

Fig. S4. Analysis of melanotrophs in *Pomc-CreERT2;p27$^{-/-}$;Rosa26$^{ReYFP/+}$* pituitaries.

A) eYFP mosaic recombination pattern in 6-month old *Pomc-CreERT2;Rosa26R$^{eYFP/+}$* and *Pomc-CreERT2;p27$^{-/-}$;Rosa26R$^{eYFP/+}$* IL. Only a proportion of melanotrophs are eYFP positive after tamoxifen treatment.

B) Histological H&E stained sections of 28 to 33-week old wt, *p27$^{-/-}$* and *Pomc-Cre;Sox2$^{fl/+;}$p27$^{-/-}$* pituitaries. *Pomc-Cre;Sox2$^{fl/+;}$p27$^{-/-}$* sections display a normal IL (Scale bar 100 microns).

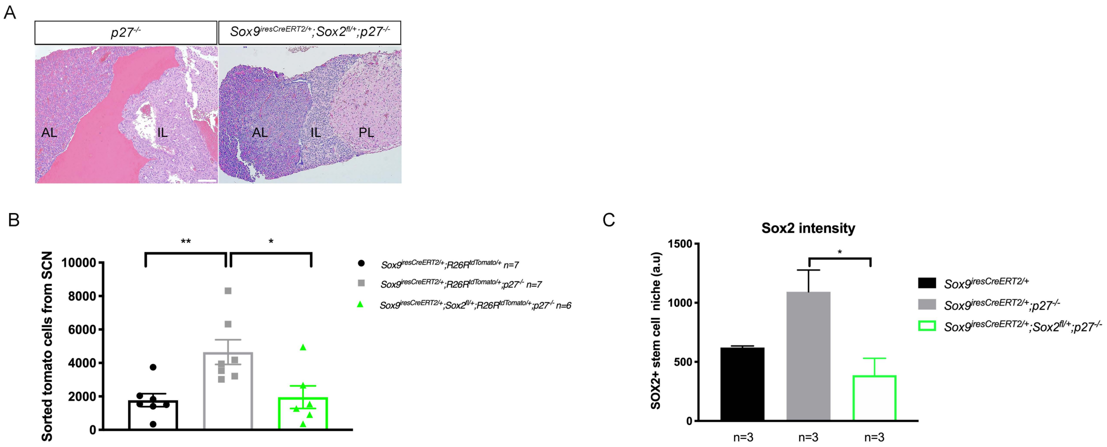

Fig. S5. Analysis of stem cells in *Sox9*$^{iresCreERT2/+}$*;Sox2*$^{fl/+}$*;p27*$^{-/-}$ pituitaries.

A) Histological H&E stained sections of *p27*$^{-/-}$ and *Sox9*$^{iresCreERT2/+}$*;Sox2*$^{fl/+}$*;p27*$^{-/-}$*;Rosa26*^{tdTomato} pituitaries. *Sox9*$^{iresCreERT2/+}$*;Sox2*$^{fl/+}$*;p27*$^{-/-}$*;Rosa26*^{tdTomato} pituitaries show a reduced IL size compared to *p27*$^{-/-}$ (Scale bar 100 microns).

B) Analysis of tomato positive cells by flow cytometry in *Sox9*$^{iCreERT2/+}$*;Rosa26*$^{tdTomato/+}$, *Sox9*$^{iCreERT2/+}$*;p27*$^{-/-}$*;Rosa26*$^{tdTomato/+}$ and *Sox9*$^{iCreERT2/+}$*;Sox2*$^{fl/+}$*;p27*$^{-/-}$*;Rosa26*$^{tdTomato/+}$ dissected IL from 3.5- to 5-month old animals that were treated by tamoxifen at 1month-old. The number of tomato-positive FACSorted stem cells is reduced in *Sox9*$^{iCreERT2/+}$*;Sox2*$^{fl/+}$*;p27*$^{-/-}$ *Rosa26*$^{tdTomato/+}$ (1953±1658, n=6) compared to *Sox9*$^{iCreERT2/+}$*;p27*$^{-/-}$*;Rosa26*$^{tdTomato/+}$ samples (4648±1954, n=7), to values comparable to control. (**p=0.0094 and *p=0.0189).

C) Quantification of SOX2 immunofluorescence intensity in the stem cell layer in *Sox9*$^{iCreERT2/+}$ (621±23 a.u, number of cells counted 1214, n=3 animals), *Sox9*$^{iCreERT2/+}$*;p27*$^{-/-}$ (1093±317 a.u, number of cells counted 899, n=3) and *Sox9*$^{iCreERT2/+}$*;Sox2*$^{fl/+}$*;p27*$^{-/-}$ (387±246 a.u, number of cells counted 1265, n=3) pituitaries of 7 to 12 month-old animals. *Sox9*$^{iCreERT2/+}$*;Sox2*$^{fl/+}$*;p27*$^{-/-}$ express lower levels of Sox2 (*p=0.02). Representative immunofluorescence is shown Fig.5F.

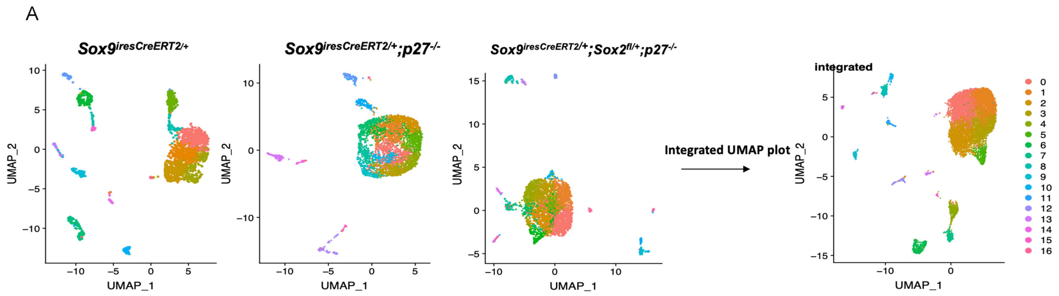

Fig. S6. Analysis of single cell RNAseq datasets from dissected ILs.

A) UMAP (Uniform Manifold Approximation and Projection) visualization of 3-month old *Sox9*$^{iresCreERT2/+}$ (n=2110 cells), *Sox9*$^{iresCreERT2/+}$*;p27*$^{-/-}$ (n=4527 cells) and *Sox9*$^{iresCreERT2/+}$*;Sox2*$^{fl/+}$*;p27*$^{-/-}$ (n=4305 cells) single cell RNAseq datasets from dissected ILs. Individual datasets were integrated on a unique UMAP plot from the three different genotypes (right panel).

Table S1. Primers for CRISPR genome editing

sgRNA	*Primer name*	*Sequence 5' to 3' (20-mer sgRNA recognition sequences in bold)*
**GUIDE 1 UPSTREAM SRR2 ENHANCER**	G1 UP SSR2_F	5’ CACCG **TCG TTT TTA GGG TAA GGT AC** 3’
	G1 UP SSR2_R	5’ AAAC GT ACC TTA CCC TAA AAA CGAC 3’
	T7 G1 UP SSR2_F	5’ TTAATACGACTCACTATAGGG G**TCG TTT TTA GGG TAA GGT AC** 3’
**GUIDE 4 UPSTREAM SRR2 ENHANCER**	G4 UP SRR2_F:	5’ CACC **GAA CCT GCC TAG CTT GGA CT** 3’
	G4 UP SRR2_R:	5’ AAAC AG TCC AAG CTA GGC AGG TTC 3’
	T7 G4 UP SSR2_F	5’ TTAATACGACTCACTATAGGG **GAA CCT GCC TAG CTT GGA CT** 3’
**GUIDE 1 DOWNSTREAM SRR2 ENHANCER**	G1 DOWN SSR2_F	5’ CACCG**AGACCCAAATGACGGGCGTG** 3’
	G1 DOWN SSR2_R	5’ AAAC CACGCCCGTCATTTGGGTCTC 3’
	T7 G1 DOWN SSR2_F	5' TTAATACGACTCACTATAGGGG**AGACCCAAATGACGGGCGTG**3’
**GUIDE 5 DOWNSTREAM SRR2 ENHANCER**	G5 DOWN SRR2_F:	5’ CACC G**TCC CCC CAC GCC CGT CAT TT** 3’
	G5 DOWN SRR2_R:	5’ AAAC AAATGACGGGCGTGGGGGGAC 3’
	T7 G5 DOWN SSR2_F	5’ TTAATACGACTCACTATAGGG G**TCC CCC CAC GCC CGT CAT TT** 3’
**pX330 plasmid**	uniR	AAAAGCACCGACTCGGTGCC

Table S1. Primers used for CRISPR genome editing

Table S2 Genotyping Primers	
Primer name	Sequence 5' to 3'
SRR2_FORWARD	5’ GGAGGATTATGAAGGTCCTG 3’
SSR2_REVERSE	5’ GCTGAGTCGGGTCAATTATT 3’
SRR2_FORWARD miSEQ (Illumina adaptors in bold)	5’**TCGTCGGCAGCGTCAGATGTGTATAAGAGACAG**GGAGGATTATGAAGGTCCTG 3’
SSR2_REVERSE miSEQ (Illumina adaptors in bold)	5’ **GTCTCGTGGGCTCGGAGATGTGTATAAGAGACAG**GCTGAGTCGGGTCAATTATT 3’

Table S2. Primers used to genotype *Srr2* deleted mice.

Table S3. Quantitative PCR Primers			
Gene Name	***Forward primer***	***Reverse Primer***	***Amplicon Size***
Pomc	CAGTGCCAGGACCTCACC	CAGCGAGAGGTCGAGTTTG	72
Pax7	GGCACAGAGGACCAAGCTC	GCACGCCGGTTACTGAAC	60
Tbx19	TGAAATGATCGTGACCAAGAACGG	TTCACCATTGACGTACTTCCAGCG	144
Sox2	AACGCCTTCATGGTATGGTC	GATCTCCGAGTTGTGCATCTT	78
Sox9	AGCTCACCAGACCCTGAGAA	CTCCAGCAATCGTTACCTTC	205

Table S3. Primers used for quantitative PCR.

Figure 3c Brightfield images		
**Genotype**	**Age (weeks)**	**Sex**
**WT**	61	male
p27-/-	52	male
p27-/-;srr2del/del	60	male

Figure 4e Brightfield images		
**Genotype**	**Age (weeks)**	**Sex**
Pomc-Cre	33	male
Pomc-Cre;p27-/-	29	male
Pomc-Cre;Sox2fl/+;p27-/-	25	male

Figure 5b Brightfield images		
**Genotype**	**Age (weeks)**	**Sex**
Sox9iresCreERT2/+;p27-/-	55	female
Sox9iresCreERT2/+;Sox2fl/+;p27-/-	53	male

Figure 3d		
**Genotype**	**Age (weeks)**	**Sex**
**WT**	33-52	4m
p27-/-	40-41	2m/2f
p27-/-;Srr2del/del	43-45	3m/1f

Figure 4b		
**Genotype**	**Age (weeks)**	**Sex**
**WT**	9	1f
	20-30	2m
p27-/-	9	1f
	20-36	2f/5m
Pomc-CreERT2;Sox2fl/+;p27-/-;EYFP+/-	9	2f
	20-30	2m

Figure 4c		
**Genotype**	**Age (weeks)**	**Sex**
Pomc-CreERT2;Sox2fl/+;p27-/-;EYFP+/-	9	2f/1m
	20-30	3m

Figure 4g-j		
**Genotype**	**Age (weeks)**	**Sex**
Pomc-Cre;p27-/-	25-43	3f/3m
Pomc-Cre;Sox2fl/+;p27-/-	25-43	1f/4m

Figure 5		
**Genotype**	**Age (months)**	**Sex**
**WT**	7 to 12	1f/5m
p27-/-	7 to 12	1f/3m
**Sox9iresCreERT2;Sox2fl/+;p27-/-**	7 to 12	3f/3m

Table S4. Information on experimental animals.

As previously reported (8), IL tumor penetrance was 100% in p27 null animal. Furthermore, no phenotypic difference was observed between males and females.

Bulk IL RNAseq (Fig. 1 C & D)			
**Genotype**	**Age**	**Sex**	**Aim**
**WT**	2 month	3 male	Define molecular pathways characteristic of pre-tumoral phenotype
**p27-/-**	2 month	1 female/1 male	
**WT**	7 month	1 female/1 male	Define molecular pathways characteristic of tumoral phenotype
**p27-/-**	7 month	1 female/2 male	

Bulk IL RNAseq (Fig. 2B)			
**Genotype**	**Age**	**Sex**	**Aim**
**WT**	2 month	2 female/2 male	
**p27-/-**	2 month	2 female/1 male	Define the pathways involved in tumorigenesis
**p27-/-;Sox2+/-**	2 month	2 female/1 male	that are modulated by *Sox2* dosage

IL sc-RNAseq (Fig. 6A)			
**Genotype**	**Age**	**Sex**	**Aim**
**Sox9iresCreERT2/+**	3 month	pool 2 males	Independent analysis of p27 null melanotrophs
**Sox9iresCreERT2/+;p27-/-**	3 month	pool male & female	and p27null;Sox2+/- stem cells to define molecular players
**Sox9iresCreERT2/+;Sox2fl/+;p27-/-**	3 month	pool male & female	involved in interactions between both cell types

Table S5. Aims and information on animals used for the transcriptomic analyses.

Dataset S1. Lists of DEG and enriched pathways in 2 and 7-month old p27$^{-/-}$ vs wt IL.

Dataset S2. Lists of DEG and enriched pathways in 2 month-old p27$^{-/-}$ vs wt and p27$^{-/-}$;Sox2$^{+/-}$ IL.

Dataset S3. List of DEG in SC clusters (Single-cell RNAseq analysis)

Genes differentially expressed in cluster 1 (comprising mostly *Sox9* $^{iresCreERT2/+}$*;p27*$^{-/-}$ cells*)* but unaffected in cluster 2 (comprising mostly *Sox9* $^{iresCreERT2/+}$*;p27*$^{-/-}$*;Sox2*$^{fl/+}$ cells) compared to cluster 0 (comprising mostly control *Sox9* $^{iresCreERT2/+}$ cells) are listed. The percentage of cells expressing the gene of interest in each cluster is represented, followed by the significance of differential expression versus control.

SI References

1. Li H*, et al.* (2012) p27(Kip1) directly represses Sox2 during embryonic stem cell differentiation. *Cell Stem Cell* 11(6):845-852.
2. Lytle NK, Barber AG, & Reya T (2018) Stem cell fate in cancer growth, progression and therapy resistance. *Nat Rev Cancer* 18(11):669-680.
3. Lamolet B*, et al.* (2001) A pituitary cell-restricted T box factor, Tpit, activates POMC transcription in cooperation with Pitx homeoproteins. *Cell* 104(6):849-859.
4. Budry L*, et al.* (2012) The selector gene Pax7 dictates alternate pituitary cell fates through its pioneer action on chromatin remodeling. *Genes Dev* 26(20):2299-2310.
5. Langlais D, Couture C, Kmita M, & Drouin J (2013) Adult pituitary cell maintenance: lineage-specific contribution of self-duplication. *Mol Endocrinol* 27(7):1103-1112.
6. Quereda V & Malumbres M (2009) Cell cycle control of pituitary development and disease. *J Mol Endocrinol* 42(2):75-86.
7. Sharma SS & Pledger WJ (2016) The non-canonical functions of p27(Kip1) in normal and tumor biology. *Cell Cycle* 15(9):1189-1201.
8. Fero ML*, et al.* (1996) A syndrome of multiorgan hyperplasia with features of gigantism, tumorigenesis, and female sterility in p27(Kip1)-deficient mice. *Cell* 85(5):733-744.
9. Kiyokawa H*, et al.* (1996) Enhanced growth of mice lacking the cyclin-dependent kinase inhibitor function of p27(Kip1). *Cell* 85(5):721-732.
10. Nakayama K*, et al.* (1996) Mice lacking p27(Kip1) display increased body size, multiple organ hyperplasia, retinal dysplasia, and pituitary tumors. *Cell* 85(5):707-720.
11. Roussel-Gervais A*, et al.* (2010) Cooperation between cyclin E and p27(Kip1) in pituitary tumorigenesis. *Mol Endocrinol* 24(9):1835-1845.
12. Bilodeau S, Roussel-Gervais A, & Drouin J (2009) Distinct developmental roles of cell cycle inhibitors p57Kip2 and p27Kip1 distinguish pituitary progenitor cell cycle exit from cell cycle reentry of differentiated cells. *Mol Cell Biol* 29(7):1895-1908.

13. Tong W & Pollard JW (2001) Genetic evidence for the interactions of cyclin D1 and p27(Kip1) in mice. *Mol Cell Biol* 21(4):1319-1328.
14. Wuebben EL & Rizzino A (2017) The dark side of SOX2: cancer - a comprehensive overview. *Oncotarget* 8(27):44917-44943.
15. Ku SY*, et al.* (2017) Rb1 and Trp53 cooperate to suppress prostate cancer lineage plasticity, metastasis, and antiandrogen resistance. *Science* 355(6320):78-83.
16. Mu P*, et al.* (2017) SOX2 promotes lineage plasticity and antiandrogen resistance in TP53- and RB1-deficient prostate cancer. *Science* 355(6320):84-88.
17. Goldsmith S, Lovell-Badge R, & Rizzoti K (2016) SOX2 is sequentially required for progenitor proliferation and lineage specification in the developing pituitary. *Development* 143(13):2376-2388.
18. Fauquier T, Rizzoti K, Dattani M, Lovell-Badge R, & Robinson IC (2008) SOX2-expressing progenitor cells generate all of the major cell types in the adult mouse pituitary gland. *Proc Natl Acad Sci U S A* 105(8):2907-2912.
19. Rizzoti K, Akiyama H, & Lovell-Badge R (2013) Mobilized adult pituitary stem cells contribute to endocrine regeneration in response to physiological demand. *Cell Stem Cell* 13(4):419-432.
20. Andoniadou CL*, et al.* (2013) Sox2(+) stem/progenitor cells in the adult mouse pituitary support organ homeostasis and have tumor-inducing potential. *Cell Stem Cell* 13(4):433-445.
21. Levy A (2002) Physiological implications of pituitary trophic activity. *J Endocrinol* 174(2):147-155.
22. Besson A, Gurian-West M, Schmidt A, Hall A, & Roberts JM (2004) p27Kip1 modulates cell migration through the regulation of RhoA activation. *Genes Dev* 18(8):862-876.
23. Borrelli E, Heyman RA, Arias C, Sawchenko PE, & Evans RM (1989) Transgenic mice with inducible dwarfism. *Nature* 339(6225):538-541.
24. Pulichino AM*, et al.* (2003) Tpit determines alternate fates during pituitary cell differentiation. *Genes Dev* 17(6):738-747.
25. Pellegata NS*, et al.* (2006) Germ-line mutations in p27Kip1 cause a multiple endocrine neoplasia syndrome in rats and humans. *Proc Natl Acad Sci U S A* 103(42):15558-15563.
26. Kelberman D*, et al.* (2006) Mutations within Sox2/SOX2 are associated with abnormalities in the hypothalamo-pituitary-gonadal axis in mice and humans. *J Clin Invest* 116(9):2442-2455.
27. Jayakody SA*, et al.* (2012) SOX2 regulates the hypothalamic-pituitary axis at multiple levels. *The Journal of clinical investigation* 122(10):3635-3646.
28. Chien WM*, et al.* (2006) Genetic mosaics reveal both cell-autonomous and cell-nonautonomous function of murine p27Kip1. *Proc Natl Acad Sci U S A* 103(11):4122-4127.
29. Furuyama K*, et al.* (2010) Continuous cell supply from a Sox9-expressing progenitor zone in adult liver, exocrine pancreas and intestine. *Nat Genet* 43(1):34-41.
30. Cheung LYM*, et al.* (2018) Single-Cell RNA Sequencing Reveals Novel Markers of Male Pituitary Stem Cells and Hormone-Producing Cell Types. *Endocrinology* 159(12):3910-3924.

31. Mayran A, *et al*. (2019) Pioneer and nonpioneer factor cooperation drives lineage specific chromatin opening. *Nature communications* 10(1):3807.
32. Haston S, *et al*. (2017) MAPK pathway control of stem cell proliferation and differentiation in the embryonic pituitary provides insights into the pathogenesis of papillary craniopharyngioma. *Development* 144(12):2141-2152.
33. Sendoel A, *et al*. (2017) Translation from unconventional 5' start sites drives tumour initiation. *Nature* 541(7638):494-499.
34. Kondoh I & Lovell-Badge R (2015) *Sox2 Biology and role in development and disease* (Elsevier).
35. Fabris L, *et al*. (2015) p27kip1 controls H-Ras/MAPK activation and cell cycle entry via modulation of MT stability. *Proc Natl Acad Sci U S A* 112(45):13916-13921.
36. Nguyen L, *et al*. (2006) p27kip1 independently promotes neuronal differentiation and migration in the cerebral cortex. *Genes Dev* 20(11):1511-1524.
37. Vilas JM, *et al*. (2015) Transcriptional regulation of Sox2 by the retinoblastoma family of pocket proteins. *Oncotarget* 6(5):2992-3002.
38. Miyagi S, *et al*. (2004) The Sox-2 regulatory regions display their activities in two distinct types of multipotent stem cells. *Mol Cell Biol* 24(10):4207-4220.
39. Marques-Torrejon MA, *et al*. (2013) Cyclin-dependent kinase inhibitor p21 controls adult neural stem cell expansion by regulating Sox2 gene expression. *Cell Stem Cell* 12(1):88-100.
40. Wang K, *et al*. (2018) FGFR1-ERK1/2-SOX2 axis promotes cell proliferation, epithelial-mesenchymal transition, and metastasis in FGFR1-amplified lung cancer. *Oncogene* 37(39):5340-5354.
41. Schaefer T & Lengerke C (2020) SOX2 protein biochemistry in stemness, reprogramming, and cancer: the PI3K/AKT/SOX2 axis and beyond. *Oncogene* 39(2):278-292.
42. Taranova OV, *et al*. (2006) SOX2 is a dose-dependent regulator of retinal neural progenitor competence. *Genes Dev* 20(9):1187-1202.
43. Srinivas S, *et al*. (2001) Cre reporter strains produced by targeted insertion of EYFP and ECFP into the ROSA26 locus. *BMC Dev Biol* 1:4.
44. Gonen N, *et al*. (2018) Sex reversal following deletion of a single distal enhancer of Sox9. *Science* 360(6396):1469-1473.
45. Rizzoti K, *et al*. (2004) SOX3 is required during the formation of the hypothalamo-pituitary axis. *Nat Genet* 36(3):247-255.
46. Barratt CLR, *et al*. (2017) The diagnosis of male infertility: an analysis of the evidence to support the development of global WHO guidance-challenges and future research opportunities. *Hum Reprod Update* 23(6):660-680.
47. Furlan A, *et al*. (2017) Multipotent peripheral glial cells generate neuroendocrine cells of the adrenal medulla. *Science* 357(6346):39-46.
48. Bankhead P, *et al*. (2017) QuPath: Open source software for digital pathology image analysis. *Sci Rep* 7(1):16878.
49. Livak KJ & Schmittgen TD (2001) Analysis of relative gene expression data using real-time quantitative PCR and the 2(-Delta Delta C(T)) Method. *Methods* 25(4):402-408.

50. McGuinness L*, et al.* (2003) Autosomal dominant growth hormone deficiency disrupts secretory vesicles in vitro and in vivo in transgenic mice. *Endocrinology* 144(2):720-731.
51. Li B & Dewey CN (2011) RSEM: accurate transcript quantification from RNA-Seq data with or without a reference genome. *BMC Bioinformatics* 12:323.
52. Dobin A*, et al.* (2013) STAR: ultrafast universal RNA-seq aligner. *Bioinformatics* 29(1):15-21.
53. Love MI, Huber W, & Anders S (2014) Moderated estimation of fold change and dispersion for RNA-seq data with DESeq2. *Genome Biol* 15(12):550.
54. Team RDC (2008) R: A language and environment for statistical computing. . *R Foundation for Statistical Computing, Vienna, Austria*.
55. Stuart T*, et al.* (2019) Comprehensive Integration of Single-Cell Data. *Cell* 177(7):1888-1902 e1821.
